# Threat gates visual aversion via theta activity in Tachykinergic neurons

**DOI:** 10.1038/s41467-023-39667-z

**Published:** 2023-07-13

**Authors:** Masato Tsuji, Yuto Nishizuka, Kazuo Emoto

**Affiliations:** 1 Department of Biological Sciences, Graduate School of Science, The University of Tokyo, 7-3-1 Hongo, Bunkyo-ku, Tokyo 113-0033 Japan; 2 International Research Center for Neurointelligence (WPI-IRCN), The University of Tokyo, 7-3-1 Hongo, Bunkyo-ku, Tokyo 113-0033 Japan

**Keywords:** Sensory processing, Cellular neuroscience

## Abstract

Animals must adapt sensory responses to an ever-changing environment for survival. Such sensory modulation is especially critical in a threatening situation, in which animals often promote aversive responses to, among others, visual stimuli. Recently, threatened Drosophila has been shown to exhibit a defensive internal state. Whether and how threatened Drosophila promotes visual aversion, however, remains elusive. Here we report that mechanical threats to Drosophila transiently gate aversion from an otherwise neutral visual object. We further identified the neuropeptide tachykinin, and a single cluster of neurons expressing it (“Tk-GAL4^2^ ∩ Vglut neurons”), that are responsible for gating visual aversion. Calcium imaging analysis revealed that mechanical threats are encoded in Tk-GAL4^2^ ∩ Vglut neurons as elevated activity. Remarkably, we also discovered that a visual object is encoded in Tk-GAL4^2^ ∩ Vglut neurons as θ oscillation, which is causally linked to visual aversion. Our data reveal how a single cluster of neurons adapt organismal sensory response to a threatening situation through a neuropeptide and a combination of rate/temporal coding schemes.

## Abstract (web summary)

A brief (maximum 250 characters, including spaces) summary; Please provide this summary in your cover letter. Animals often promote visual aversion in a threatening situation for survival. Tsuji et al. report in Drosophila that a single cluster of Tachykinergic neurons translate mechanical threats to gating of visual aversion through θ oscillation.

## Introduction

The capability of adapting sensory responses to an ever-changing environment, especially when the situation is threatening, is crucial for survival. Threatened animals not only show escape responses and cardiac reactions^1–5^, but also drastically modulate a wide variety of sensory responses including nociception^6^, audition^7^, and vision^8–11^. Of these, vision provides the richest source of information for diurnal animals about the external world, and hence its modulation in a threatening situation likely plays a vital role. Indeed, it has widely been documented that threat often acts to promote visual aversion^8–11^.

Animal studies have partially revealed neuronal mechanisms by which threat promotes visual aversion. In fish, for instance, sound pip that itself evokes a startle response also enhances a startle response to a visual loom^8^. This regulation may be achieved by the excitatory convergence of mechanical and visual inputs downstream of the optic tectum^8,12^. In addition to excitatory convergence, visual circuits such as the primary visual cortex^13^, lateral geniculate nucleus^14,15^, superior colliculus^16^ and even retinal cells^16^ or equivalent of them^17,18^ are known to be under the strong influence of neuromodulation^19,20^. However, how threat might regulate visual responses – specifically, the brain structure regulated by threat and the molecular, possibly neuromodulatory, mechanism of such regulation – remains so far largely unknown.

Drosophila offers an attractive model in tackling these problems. First, flies are equipped with a sophisticated and well-studied visual system that in many ways parallels with that of mammals in terms of anatomy and behaviors it elicits^21–26^. It is also under the influence of a rich repertoire of modulation^20^. One of the best known examples is the effect of locomotion^27–30^ conserved across species^31,32^, but others include the effects of food odor^17^ and sexual arousal^18^. While known forms of visual neuromodulation are based on a functional equivalent of noradrenaline named octopamine^17,29,33^, Drosophila harbors many more evolutionarily conserved neuromodulators^34^. Last, threatened Drosophila exhibits signs of a threat-induced internal state. Specifically, flies exposed to visual threats show running, jumping, freezing, and suppression of feeding^35^. Similarly, flies exposed to a series of air puffs increase locomotion and olfactory startle response, with the locomotion being regulated by Dop1R1^36^. In addition, flies presented with a visual loom show bradycardia with freezing and tachycardia with flight responses^5^. Such existing knowledge led us to hypothesize that threat might promote visual aversion through a neuromodulatory mechanism in Drosophila, which would provide an appealing opportunity to dissect the underlying molecular and neuronal mechanisms.

Here we report that air puffs, which we confirmed to elicit signs of an internal state in Drosophila, transiently gate aversion from an otherwise neutral, small visual object. We further identified the neuropeptide tachykinin, and a single cluster of neurons expressing it (“Tk-GAL4^2^ ∩ Vglut neurons”), that are responsible for gating visual aversion. Calcium imaging experiments revealed that Tk-GAL4^2^ ∩ Vglut neurons encode the air puffs as increased activity. Unexpectedly, we discovered that these neurons encode a visual object as increased θ oscillation only when air puffs are given beforehand, which is causally linked to visual aversion. Altogether, our data identify Tk and a cluster of Tk-expressing neurons that gate visual aversion in threatened Drosophila, and illustrate the coding schemes therein to adapt organismal sensory responses to a threatening situation.

## Results

### Air puffs increase locomotion and gate visual aversion

**Fig. 1:**
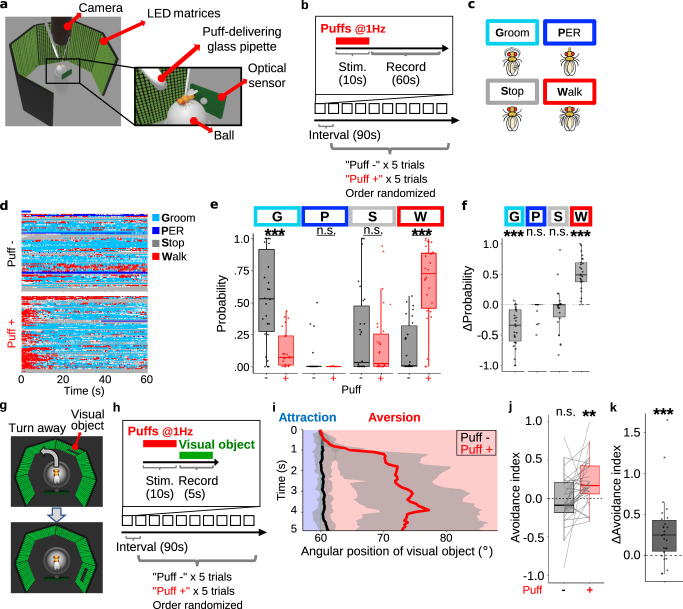
Air puffs promote escape behaviors and visual aversion. **a** A schematic illustration of an LED arena. **b** Time course of the experiment. **c** Analyzed behaviors (grooming, proboscis extension reflex (PER), stopping, and walking). **d** Ethogram representing the time course of behaviors. Light blue, dark blue, gray, and red areas indicate grooming, PER, stopping, walking, respectively. *N* = 26. **e** Probability of each behavior per fly during the initial 5 s in **d**. In this and following, box plots are generated so that center line indicates median, box limits indicate upper and lower quartiles, and whiskers indicate 1.5x interquartile range. *N* = 26, ***p < 0.001, n.s.: *p* > 0.05, two-tailed *t*-test or Wilcoxon signed rank test followed by Benjamini–Hochberg correction. **f** Puff-induced changes in the probability of each behavior in **e**. Statistics are identical to those in **e**. **g** A schematic of the experiment. A small visual object was displayed on a set of LED matrices surrounding the fly. The fly’s walking speed and linear direction along *x*-axis were continuously monitored and were reflected on the visual object’s angular position. This schematic illustrates how the fly’s left-ward walking results in the right-ward shift in the visual objects’ angular position. **h** Time course of the experiment. **i** Example time course of the visual object’s angular position. Results of all trials of an example fly were pooled. Black line indicates “puff -“ trials, whereas red line indicates “puff +“ trials. Lines and shaded areas represent mean and SEM, respectively. Sky-blue vertical line indicates 60 degrees at which the visual object initially appeared. **j** Average avoidance indices of “puff -“ trials and “puff +“ trials per fly. *N* = 26, **p < 0.01, n.s.: *p* > 0.05, two-tailed *t*-test followed by Bonferroni correction. **k** Changes in the avoidance indices presented in **j**. *N* = 26, ***p < 0.001, two-tailed Wilcoxon signed rank test.

For Drosophila, a small visual object could signal the presence of either a potential mate or a distant predator, and hence its ethological value presumably depends on the context. Consistently, a small object usually induces no response^37^ or only mild aversion^38^, but induces attraction when combined with food odor^17^ or sexual arousal^18^. We thus hypothesized that aversion from a small object is promoted in a threatening situation. To test this possibility, we took advantage of a walking simulator in which it is easy to present air puffs as mechanical threats and a visual object at a location and a timing of our choosing (Fig. 1a). Since previous studies reported increased locomotion as one of the primary manifestations of a threat-induced internal state^35,36^, we first sought to confirm that air puffs indeed increase locomotion in our paradigm (Fig. 1b). When we estimated the fly’s walking velocity through the ball’s movement (Fig. 1a), we found that the fly’s walking velocity instantaneously increased upon air puffs (500 ms puff at 1 Hz for 10 times), which then gradually returned to the baseline (Supplementary Fig. 1a), consistent with the previous report^36^. Also consistently^36^, increasing the number of puffs tended to increase the maximum walking velocity (Supplementary Fig. 1b). To better characterize the puff-induced behavioral changes, we quantified grooming, stopping, proboscis extension reflex (PER), and walking behaviors with our custom-written convolutional neural network software (Fig. 1c, Supplementary Fig. 1c; see Online Methods). This analysis revealed that grooming and stopping dominated before air puffs (Fig. 1d, upper panel). After puffs, however, these behaviors were taken over by walking (Fig. 1d, lower panel), especially during the initial 5 s (Fig. 1d, blue bar above the upper panel). This observation was echoed when we quantified the probability of each behavior during the initial 5 s of the recordings (Fig. 1e), along with their per-fly differences between puff - and puff + trials (Fig. 1f). Moreover, in the closed-loop experiment in which air puffs were applied only when the fly was heading in certain directions, flies clearly avoided heading in the “puff-ON” directions (Supplementary Fig. 1d-f). These observations reinforce the idea that air puffs serve as mechanical threats to flies^25^.

We next tested whether air puffs gate visual aversion. To this end, we confronted the fly with a small visual object, initially 60 degrees right or left to the fly at random, that shifts its position according to the fly’s walking pattern (closed-loop) (Fig. 1g). A single trial consisted of 90 s of interval, 10 s of air puffs, and 5 s of recording, and was repeated for each fly 10 times with or without air puffs at random (Fig. 1h). When we tracked the position of a visual object as the fly walked without preceding puffs, directed walking toward or away from the visual object was not evident (Fig. 1i, black line; Supplementary Movie 1). In stark contrast, when a small object was presented following air puffs, flies clearly shifted the visual object to the fly’s back (Fig. 1i, red line; Supplementary Movie 1). This effect, however, may simply be because increased locomotion amplifies the aversion which is too weak to detect without preceding puffs. To address this issue, we calculated the avoidance index, as defined by the linear distance travelled away from the object, divided by the total linear distance travelled (see Online Methods). This index increased following the air puffs (Fig. 1j), suggesting that the gated aversion is unaffected by increased locomotion. Since the effect of air puffs was clearly summarized as positive per-fly changes in visual aversion (Fig. 1k), the following data are presented in this format. Gated visual aversion was not affected by repeated trials (Supplementary Fig. 1g) nor sex-dependent at a statistically significant level (Supplementary Fig. 1h), persisted for up to 6 s but no longer than 10 s (Supplementary Fig. 1i), and required more than 5 puffs to manifest (Supplementary Fig. 1j). We thus established that air puffs serve as mechanical threats for Drosophila to gate aversion from a small visual object.

### Air puffs gate visual aversion via neuropeptide Tachykinin

**Fig. 2:**
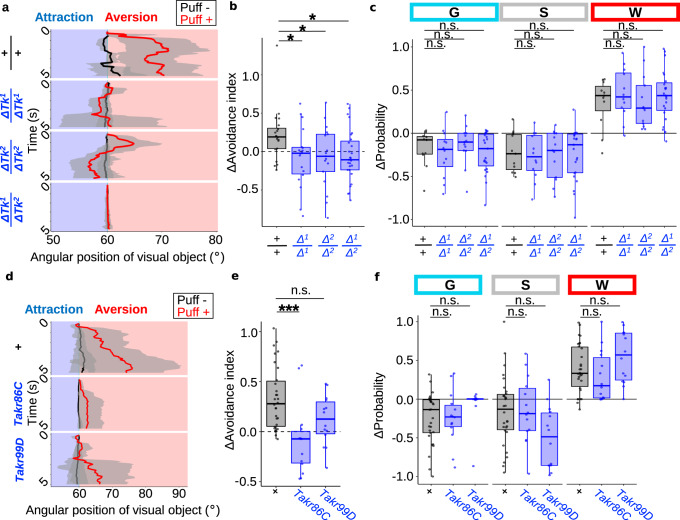
Tk is required for puff-gated visual aversion but not for increased locomotion. **a** Example time course of the visual object’s angular position for wild-type and *ΔTk* (*ΔTk*^*1*^ and *ΔTk*^*2*^) homo- and trans-heterozygous mutants. For each genotype, results of all trials of an example fly were pooled. Lines and shaded areas represent means and SEM, respectively. **b** Puff-induced changes in the avoidance indices. *N* = 20, 18, 17, 27, for WT, *ΔTk*^*1*^, *ΔTk*^*2*^, *ΔTk*^*1*^/*ΔTk*^*2*^, respectively. In this and following, box plots are generated so that center line indicates median, box limits indicate upper and lower quartiles, and whiskers indicate 1.5x interquartile range. *p < 0.05, two-tailed Wilcoxon rank sum test followed by Benjamini–Hochberg correction. Genotypes are shown in abbreviated forms (e.g. “*Δ*^*1*^” instead of “*ΔTk*^*1*^”). **c** Puff-induced changes in the probability of each behavior. *N* = 20, 18, 17, 27, for WT, *ΔTk*^*1*^, *ΔTk*^*2*^, *ΔTk*^*1*^/*ΔTk*^*2*^, respectively. n.s.: *p* > 0.05, two-tailed *t*-test or Wilcoxon rank sum test followed by Benjamini–Hochberg correction. **d** Example time course of the visual object’s angular position for wild-type and homozygous mutants of Takr subtypes. Lines and shaded areas represent means and SEM, respectively. **e** Puff-induced changes in the avoidance indices. *N* = 30, 14, 14, for WT, Takr86C, Takr99D, respectively. ***p < 0.001, n.s.: *p* > 0.05, two-tailed Wilcoxon rank sum test followed by Bonferroni correction. **f** Puff-induced changes in the probability of each behavior. *N* = 20, 18, 17, 27, for WT, *ΔTk*^*1*^, *ΔTk*^*2*^, *ΔTk*^*1*^/*ΔTk*^*2*^, respectively. n.s.: *p* > 0.05, two-tailed *t*-test or Wilcoxon rank sum test followed by Benjamini–Hochberg correction.

To probe the molecular mechanism underlying the puff-gated visual aversion, we screened a set of CRISPR-generated neuropeptide null mutants^39^. This identified a few lines, the most prominent of which was the mutant line of Tachykinin (Tk), which failed to gate visual aversion while increasing the locomotion (Supplementary Data 1). To further validate the necessity of Tk, we tested additional null mutations (*ΔTk*^*1*^ and *ΔTk*^*2*^) reported previously^40^. As expected, puff-gated visual aversion was suppressed in homozygous mutants of *ΔTk*^*1*^ (*ΔTk*^*1*^/*ΔTk*^1^) and *ΔTk*^2^ (*ΔTk*^2^/*ΔTk*^2^), as well as their trans-heterozygotes (*ΔTk*^1^/*ΔTk*^2^) (Fig. 2a, b), further supporting the role of Tk in gating visual aversion. Interestingly, increased locomotion upon air puffs did not significantly differ between wild-type and these mutants (Fig. 2c), indicating that Tk is specifically required for gating visual aversion but not for increased locomotion. Tk mutants showed optomotor response (Supplementary Fig. 2a-c) and aversion from air puffs (Supplementary Fig. 2d, e) to the extent comparable to those of the wild-type flies, suggesting that these mutants retain basic visual/motor functions. To obtain further evidence that Tk is required for gating visual aversion, we tested mutants of Tk receptors. In the fly genome, Takr86C^41^ and Takr99D^42^ have been identified. We thus tested a putative loss-of-function insertional mutation in Takr99D^43^ and a loss-of-function deletional mutation in Takr86C^40^. Puff-gated visual aversion was suppressed in Takr86C mutant (Fig. 2d, e), phenocopying the Tk mutations (Fig. 2a, b). Similar tendency was observed for Takr99D mutant, but not at a statistically significant level (Fig. 2a, b). Also consistent with the phenotype of Tk mutations (Fig. 2c), both Takr mutants did show increased locomotion (Fig. 2f). These data corroborate the idea that Tk is specifically required for gating visual aversion but not for increased locomotion.

### Air puffs gate visual aversion via Tk^+^ neurons

**Fig. 3:**
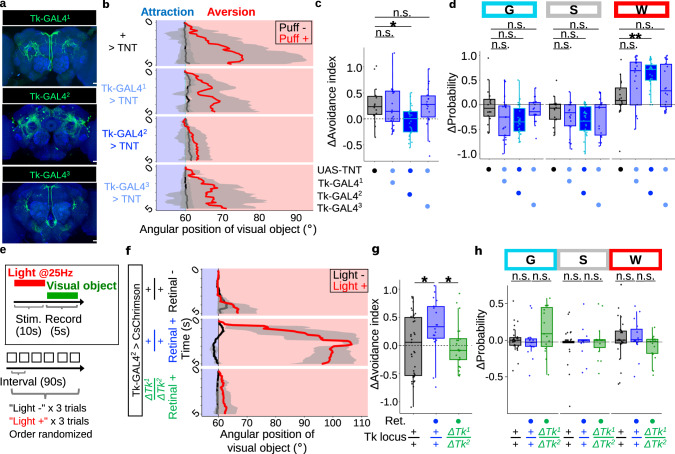
Neurons expressing Tk are responsible for gated visual aversion but not for increased locomotion. **a** Brains of Tk-GAL4^1^ > mCD8::GFP, Tk-GAL4^2^ > mCD8::GFP, and Tk-GAL4^3^ > mCD8::GFP (green) immunostained with anti-neuropil marker nc82 (blue). Scale bars: 25 um. Similar results were obtained across 4 independent samples. **b** Example time courses of the visual object’s angular position for +>TNT (effector control), Tk-GAL4^1^ > TNT, Tk-GAL4^2^ > TNT, Tk-GAL4^3^ > TNT. For each genotype, results of all trials of an example fly were pooled. Lines and shaded areas represent means and SEM, respectively. **c** Puff-induced changes in the avoidance indices. *N* = 16, 21, 27, 19, for +>TNT, Tk-GAL4^1^ > TNT, Tk-GAL4^2^ > TNT, Tk-GAL4^3^ > TNT, respectively. In this and following, box plots are generated so that center line indicates median, box limits indicate upper and lower quartiles, and whiskers indicate 1.5x interquartile range. *p < 0.05, n.s.: *p* > 0.05, two-tailed *t*-test followed by Benjamini–Hochberg correction. **d** Puff-induced changes in the probability of each behavior. *N* = 16, 21, 27, 19, for +>TNT, Tk-GAL4^1^ > TNT, Tk-GAL4^2^ > TNT, Tk-GAL4^3^ > TNT, respectively. **p < 0.01, n.s.: *p* > 0.05, two-tailed *t*-test or Wilcoxon rank sum test followed by Benjamini–Hochberg correction. **e** A schematic of photoactivation experiments. Red LED light was shined to the head of a fly to activate CsChrimson driven by Tk-GAL4^2^. Each fly underwent 6 trials of “light -“ and “light +“ (3 trials each) in a randomized order. **f** Example time course of the visual object’s angular position for retinal - control group, retinal + test group, and retinal + group in the genetic background of *ΔTk*^*1*^/*ΔTk*^*2*^. For each group, results of all trials of an example fly were pooled. Lines and shaded areas represent means and SEM, respectively. **g** Light-induced changes in the avoidance indices. *N* = 35, 18, 21, for retinal -, retinal +, retinal + in the background of *ΔTk*^*1*^/*ΔTk*^*2*^, respectively. *p < 0.05, two-tailed *t*-test followed by Bonferroni correction. **h** Light-induced changes in the probability of each behavior. *N* = 35, 18, 21, for retinal -, retinal +, retinal + in the background of *ΔTk*^*1*^/*ΔTk*^*2*^, respectively. n.s.: *p* > 0.05, two-tailed *t*-test or Wilcoxon rank sum test followed by Benjamini–Hochberg correction.

We next sought to identify the Tk-expressing neurons responsible for gating visual aversion. To this end, we expressed the tetanus toxin (TNT) using GAL4 drivers under the control of different Tk promoters, with each leading to partially overlapping but different expression patterns^44^ (Fig. 3a). We found that puff-gated visual aversion was suppressed in flies in which TNT was expressed by one of these drivers (Tk-GAL4^2^) but not the other drivers, suggesting that this line labels the responsible neurons (Fig. 3b, c; Supplementary Fig. 3a). Expressing EGFP and RedStinger (nuclear RFP) with Tk-GAL4^2^ labeled a collection of approximately 50 neurons dispersed throughout the brain (Supplementary Fig. 3b). Suppression of Tk-GAL4^2^ neurons failed to disrupt the increased locomotion (Fig. 3d; Supplementary Fig. 3c), suggesting that Tk-GAL4^2^ neurons are required for gated visual aversion but not for increased locomotion. Driving Tk RNAi by Tk-GAL4^2^ resulted in the tendency to suppress puff-gated visual aversion but not quite reaching statistical significance, possibly due to insufficient efficiency (Supplementary Fig. 3d). Next, we asked whether artificial activation of these neurons can substitute for air puffs. To this end, we activated Tk-GAL4^2^ neurons by shining light on the head of the fly expressing the light-gated cation channel CsChrimson (Fig. 3e). A single trial consisted of 90 s of interval, 10 s of photoactivation, and 5 s of recording (visual object was presented soon after the photoactivation ended), and was repeated for each fly 6 times with or without shining light at random. Indeed, photoactivation of Tk-GAL4^2^ neurons alone gated visual aversion (Fig. 3f, upper two panels). This observation was echoed when we calculated the difference in the avoidance indices between light + trials and light - trials (Fig. 3g, left two plots). Importantly, this activation effect failed to manifest in the Tk-KO background (Fig. 3f, the bottom panel; Fig. 3g, the right plot), suggesting that photoactivation of Tk-GAL4^2^ neurons gated visual aversion via Tk. Consistent with the data of Tk mutants (Fig. 2) and suppression of Tk-GAL4^2^ neurons (Fig. 3a-d), photoactivation of Tk-GAL4^2^ neurons failed to increase locomotion at a statistically significant level (Fig. 3h). These data collectively suggest that Tk-GAL4^2^ neurons are necessary and sufficient for gating visual aversion but not for increased locomotion, and that these neurons exert such a function via Tk.

### A single cluster of Tk^+^ neurons gates visual aversion

**Fig. 4:**
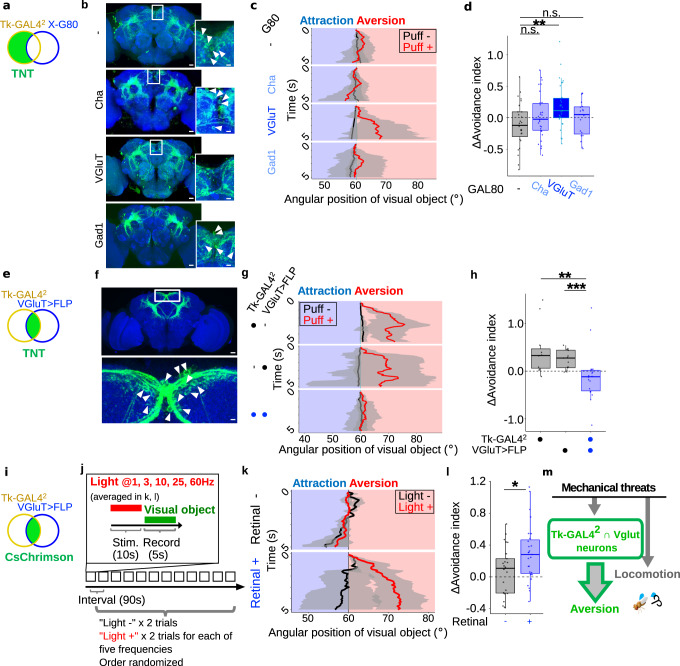
A single cluster of Tk^+^ neurons are required for air puffs to gate visual aversion. **a** A schematic diagram of combining Tk-GAL4^2^ and X-GAL80 to restrict the expression of GAL4. **b** A brain of Tk-GAL4^2^ > mCD8::GFP (green), and brains of Tk-GAL4^2^ > mCD8::GFP (green) combined with VGluT-GAL80, ChAT-GAL80, and Gad1-GAL80, respectively. In this and following, precise genotypes are available in Supplementary Data 3. Brains were immunostained with anti-neuropil marker nc82 (blue). Arrowheads in inlets indicate cell bodies. Scale bars: 25 um (inlet: 10 um). Similar results were obtained across 4, 4, 8, 5 independent samples, for GAL80 = -, Cha, VGluT, and Gad1, respectively. **c** Example time courses of the visual object’s angular position. In this and following plots **g**, **k** results of all trials of an example fly were pooled for each group, and lines and shaded areas represent means and SEM, respectively. **d** Puff-induced changes in the avoidance indices. *N* = 27, 30, 30, 19, for GAL80 = -, Cha, VGluT, and Gad1, respectively. In this and following, box plots are generated so that center line indicates median, box limits indicate upper and lower quartiles, and whiskers indicate 1.5x interquartile range. **p < 0.01, n.s.: *p* > 0.05, two-tailed *t*-test followed by Benjamini–Hochberg correction. **e** A schematic diagram of combining Tk-GAL4^2^>stop>TNT and VGluT-FLP to restrict the expression of GAL4. **f** A brain of VGluT>FLP and Tk-GAL4^2^>stop>mCD8::GFP (green) immunostained with anti-nc82 (blue). The lower image shows a magnified view of the white box in the upper image. Cell bodies are indicated with arrowheads. Scale bars: 15 um (magnification: 5 um). Similar results were obtained across 5 independent samples. **g** Example time courses of the visual object’s angular position. **h** Puff-induced changes in the avoidance indices. *N* = 11, 15, 18, for Tk-GAL4^2^>stop>TNT, VGluT>FLP, and Tk-GAL4^2^>stop>TNT & VGluT>FLP, respectively. ***p < 0.001, **p < 0.01, two-tailed Wilcoxon rank sum test followed by Bonferroni correction. **i** A schematic diagram of combining Tk-GAL4^2^>stop>CsChrimson and VGluT-FLP to restrict the expression of GAL4. **j** Time course of the experiment. **k** Example time courses of the visual object’s angular position. **l** Puff-induced changes in the avoidance indices. *N* = 25, 27 for retinal -, retinal +, respectively. *p < 0.05, two-tailed *t*-test. **m** Model of the mechanism underlying puff-gated visual aversion and increased locomotion.

We further sought to narrow down the subset of Tk-GAL4^2^ neurons that is responsible for gating visual aversion. A growing body of evidence suggests that neuropeptidergic neurons typically co-express one or more additional small neurotransmitters, including Tk-expressing neurons in the Drosophila brain^45^. We thus reasoned that combining Tk-GAL4^2^ with a GAL4 suppressor GAL80^46^, driven by markers of small neurotransmitters (Fig. 4a), may label different subsets of Tk-GAL4^2^ neurons. Indeed, this strategy resulted in the labelling of partially overlapping but distinct subpopulations of Tk-GAL4^2^ neurons (Fig. 4b). Most notably, GAL80 driven by a marker for glutamatergic neurons (VGluT), but not markers for cholinergic (ChAT) or GABAergic (Gad1) neurons, suppressed labeling of a cluster of neurons in the superior medial protocerebrum (Fig. 4b, white arrowheads in the inlets indicate cell bodies). Remarkably, VGluT-GAL80 restored the puff-gated visual aversion disrupted by Tk-GAL4^2^-driven TNT (Fig. 4c, d), suggesting that VGluT-GAL80 labels a subset of Tk-GAL4^2^ neurons responsible for gating visual aversion. VGluT-GAL80 by itself failed to influence puff-gated visual aversion, as flies carrying VGluT-GAL80 in addition to either Tk-GAL4^2^ or UAS-TNT exhibited puff-gated visual aversion comparable to flies carrying either Tk-GAL4^2^ or UAS-TNT alone (Supplementary Fig. 4a). All test groups showed increased locomotion comparable to that of the control group (Supplementary Fig. 4b), corroborating our earlier finding (Fig. 3d, h) that Tk-GAL4^2^ neurons are dispensable for puff-increased locomotion.

In order to more directly probe the function of VGluT^+^ subset of Tk-GAL4^2^ neurons, we next took advantage of the intersectional strategy^47^ in which the GAL4/ UAS binary system was combined with the Flippase (FLP) recombination technique. With this strategy, we aimed to restrict the expression of TNT to the cells in which both Tk-GAL4^2^ and VGluT-FLP are active (Fig. 4e, green area). Immunohistochemistry revealed that the intersection of Tk-GAL4^2^ and VGluT indeed labels a single cluster of 20-30 neurons in the superior medial protocerebrum (Fig. 4f, white arrowheads in the inlet indicate cell bodies), a pattern complementary to that of the combination of Tk-GAL4^2^ and VGluT-GAL80 (Fig. 4b). Hereafter, we designate these neurons as Tk-GAL4^2^ ∩ Vglut neurons. Immunohistochemistry of Tk-GAL4^2^ ∩ Vglut neurons in females revealed distinct but partially overlapping neurite morphology compared to males (Supplementary Fig. 4c). To examine whether Tk-GAL4^2^ ∩ Vglut neurons are required for puff-gated visual aversion, we next expressed TNT in these neurons. This manipulation suppressed puff-gated visual aversion compared with control groups (Fig. 4g, h), suggesting that Tk-GAL4^2^ ∩ Vglut neurons are required for gating visual aversion.

Next, since artificial induction of Tk-GAL4^2^ neurons substituted for the air puffs in gating visual aversion (Fig. 3e–g), we expected to obtain a similar result for Tk-GAL4^2^ ∩ Vglut neurons. Indeed, when we photoactivated the Tk-GAL4^2^ ∩ Vglut neurons expressing CsChrimson in the same manner as in Fig. 3e–g (Fig. 4i, j), visual aversion was gated in a retinal-fed group but not in a retinal-unfed control group (Fig. 4k, l). Photoactivation of Tk-GAL4^2^ ∩ Vglut neurons failed to increase locomotion at a statistically significant level (Supplementary Fig. 4e), suggesting the specific role of these neurons in gating visual aversion. Varying the photoactivation frequency failed to influence the photoactivation effect on visual aversion or locomotion at statistically significant levels (Supplementary Fig. 4f, g), suggesting that increased activity in Tk-GAL4^2^ ∩ Vglut neurons but not the temporal pattern promotes visual aversion. Thus, photoactivation of Tk-GAL4^2^ ∩ Vglut neurons overall phenocopied photoactivation of Tk-GAL4^2^ neurons (Fig. 3e–g). Collectively, our data suggest that the activation of Tk-GAL4^2^ ∩ Vglut neurons is causally related to the puff-gated visual aversion, but is not causally related to increased locomotion (Fig. 4m).

### Tk-GAL4^2^ ∩ Vglut neurons are activated by air puffs

**Fig. 5:**
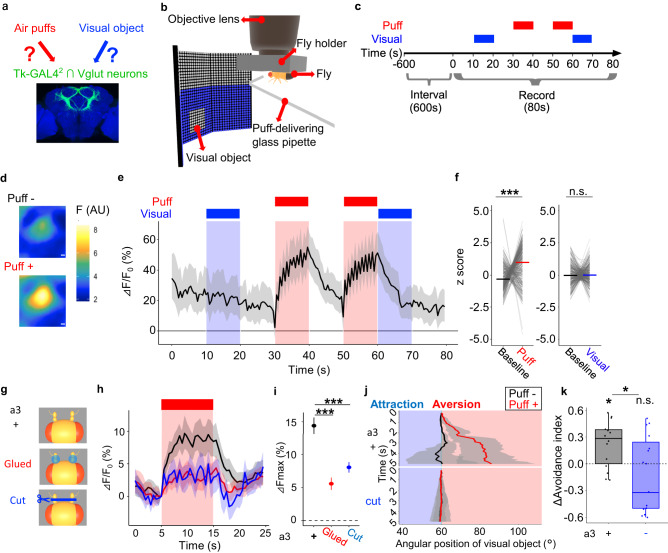
Tk-GAL4^2^ ∩ Vglut neurons are activated by air puffs. **a** A schematic of the hypothesis. Scale bar: 15 um. **b** A schematic of the setup for calcium imaging. **c** Time course of the experiment. Each fly underwent 3–5 recordings. **d** Pseudocolour image of the fluorescent changes in soma of an example Tk-GAL4^2^ ∩ Vglut neuron upon air puffs. Scale bars: 1 um. **e** Time course of the ΔF/F_0_ (%). In this and following **h**, **j**, lines and shaded regions represent means and SEM, respectively. *N* = 187 cells from 6 flies. **f** Calcium response evoked by puffs and visual stimulus. The maximal z-scored ΔF/F_0_ values after stimulus onset were averaged. Baseline represents ΔF/F_0_ of the frame immediately prior to stimulus onset. Lines represent medians. ***p < 0.001, n.s.: *p* > 0.05, two-tailed *t*-test or Wilcoxon signed rank test followed by Bonferroni correction. *N* = 329 cells from 11 flies. Results of “forward” schedule illustrated in **c** and “reverse” schedule in which air puffs and visual stimulus were presented in the reverse order were pooled. **g** a3 segment of both antennae were incapacitated by either gluing (“Glued”) or surgical removal (“Cut”). **h** Time courses of the ⊿F/F_0_ (%) for each group. *N* = 217, 96, 152 cells from 17, 8, 13 animals for “+“, “Glued”, “Cut”, respectively. **i** Peak ⊿F/F_0_ during air puffs from **h**. ***p < 0.001, two-tailed *t*-test followed by Bonferroni correction. Dots and error bars represent means and SEM. *N* = 217, 96, 152 cells from 17, 8, 13 animals for “+“, “Glued”, “Cut”, respectively. **j** Example time courses of the visual object’s angular position. Results of all trials of an example fly were pooled for each group. **k** Puff-induced changes in the avoidance indices. *N* = 14, 15, for a3 +, a3 cut, respectively. Box plots are generated so that center line indicates median, box limits indicate upper and lower quartiles, and whiskers indicate 1.5x interquartile range. *p < 0.05, n.s.: *p* > 0.05, two-tailed *t*-test or Wilcoxon rank sum test followed by Bonferroni correction for each group, two-tailed Wilcoxon rank sum test for between-group comparison.

We next asked whether and how Tk-GAL4^2^ ∩ Vglut neurons encode air puffs and a visual object (Fig. 5a). To this end, we imaged activity of Tk-GAL4^2^ neurons expressing GCaMP7f in a tethered fly, while presenting air puffs and a visual object (Fig. 5b). To record the neuronal response to either a visual object alone, air puffs alone, or air puffs followed by a visual object, we tested these three conditions with intervals for the same fly (Fig. 5c). This experiment revealed that Tk-GAL4^2^ ∩ Vglut neurons exhibit a calcium elevation upon air puffs (Fig. 5d; Supplementary Movie 2). More specifically, the GCaMP signal kept rising while air puffs were repeatedly applied (Fig. 5e, red-shaded time windows (30-40 s and 50-60 s)), began to decline when puff application ceased, and returned to the baseline in approximately 10 s (Fig. 5e, 40-50 s). Our recording captured signals from Tk-GAL4^2^ ∩ Vglut neurons alone, as evidenced by the lack of baseline fluorescence in our region-of-interest when VGluT-GAL80 was additionally expressed (Supplementary Fig. 5a). In contrast to air puffs, we failed to detect changes in the GCaMP signal in response to a visual object with or without preceding air puffs (Fig. 5e, blue-shaded time windows). When GCaMP signals were transformed into z-scores and averaged over the time windows for air puffs or a visual object (Fig. 5f), we observed an increase in the z-score by approximately 1.3 during air puffs (Fig. 5f, left), while we failed to observe such increase during the presentation of a visual object (Fig. 5f, right). Calcium elevation upon air puffs was also observed in females (Supplementary Fig. 5b, c). Tk-GAL4^2^ ∩ Vglut neurons thus appear to encode air puffs in the form of calcium elevation but not a visual object. The puff response was heterogeneous within Tk-GAL4^2^ ∩ Vglut neurons, with each neuron showing distinct response dynamics in a reproducible manner (Supplementary Fig. 5d). Furthermore, a subpopulation (~36%) of Tk-GAL4^2^ ∩ Vglut neurons failed to exhibit a calcium elevation upon air puffs (Supplementary Fig. 5e), without obvious spatial localization pattern stereotyped across flies (Supplementary Fig. 5f).

We next wondered from where the information of air puffs is transmitted to Tk-GAL4^2^ ∩ Vglut neurons. Since wind is sensed in part by the a3 segment of the antennae^48^, we hypothesized that disrupting the antennae function may block the response of Tk-GAL4^2^ ∩ Vglut neurons to air puffs. To test this hypothesis, we disrupted the function of a3 by either surgical removal or gluing (Fig. 5g), and imaged the calcium dynamics in Tk-GAL4^2^ ∩ Vglut neurons while delivering air puffs. Indeed, disruption of a3 markedly reduced the calcium elevation in Tk-GAL4^2^ ∩ Vglut neurons (Fig. 5h). Further quantification revealed that disruption of a3 reduced the maximum GCaMP signals during air puffs by approximately 60% (Fig. 5i). Since we have already established a causal link between Tk-GAL4^2^ ∩ Vglut neurons and gated visual aversion (Fig. 4), reduction in the puff response of Tk-GAL4^2^ ∩ Vglut neurons should lead to a decrease in gated visual aversion. In line with this reasoning, surgical removal of a3 segments suppressed the puff-gated visual aversion (Fig. 5j, k). These data indicate that air puffs activate Tk-GAL4^2^ ∩ Vglut neurons via the a3 segments of the antennae to gate visual aversion.

### Air puffs gate theta response to a visual object in Tk-GAL4^2^ ∩ Vglut neurons, which causally contributes to visual aversion

**Fig. 6:**
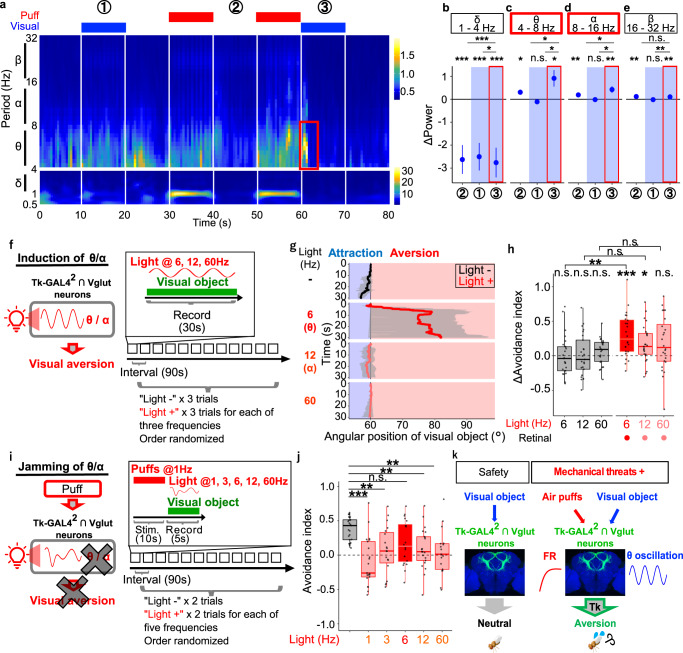
Air puffs gate theta response to a visual object in Tk-GAL4^2^ ∩ Vglut neurons, which causally contributes to visual aversion. **a** Average wavelet cross-spectrum of the neural recordings. Red rectangle indicates the puff-gated visual response in θ/ α frequencies. Distinct scales of color codes are employed for frequencies below and above 4 Hz, as air puffs applied at 1 Hz caused exceedingly strong signal around 1 Hz, hampering the visibility of the other signals. *N* = 187 cells from 6 flies. **b**–**e** Power of δ, θ, α, β oscillatory activities (averaged across frequencies within the corresponding band), subtracted by power of the corresponding band during the 0.5 s time bin immediately prior to each time window, for each cell. *N* = 151 cells from 10 flies. ***p < 0.001, **p < 0.01, *p < 0.05, n.s.: *p* > 0.05. Dots and error bars represent means and SEM. Two-tailed *t*-test followed by Benjamini–Hochberg correction for each response characteristic, and two-tailed paired *t*-test followed by Bonferroni correction for comparisons between post-puff visual window and the other windows. **f** Left: A schematic of the hypothesis. Right: Time course of the experiment. Each fly underwent 12 trials in a randomized order. **g** Example time courses of the visual object’s angular position. For each photoactivation frequency, results of all trials of an example fly were pooled. Lines and shaded areas represent means and SEM, respectively. **h** Photoinduced changes in the avoidance indices. *N* = 25, 25, for retinal -, retinal +, respectively. In this and following, box plots are generated so that center line indicates median, box limits indicate upper and lower quartiles, and whiskers indicate 1.5x interquartile range. ***p < 0.001, **p < 0.01, *p < 0.05, n.s.: *p* > 0.05, two-tailed *t*-test followed by Benjamini–Hochberg correction. **i** Left: A schematic of the hypothesis. Right: Time course of the experiment. Each fly underwent 12 trials in a randomized order. **j** Avoidance indices. *N* = 23. ***p < 0.001, **p < 0.01, n.s.: *p* > 0.05, two-tailed paired *t*-test followed by Benjamini–Hochberg correction. **k** Model of the mechanism underlying puff-gated visual aversion. Scale bar: 15 um.

Our data so far have demonstrated that air puffs but not a visual object increase the activity of Tk-GAL4^2^ ∩ Vglut neurons to gate visual aversion (Fig. 5e, f). How, then, is increased activity of Tk-GAL4^2^ ∩ Vglut neurons translated into visual aversion? Multiple information can be encoded, for example in the hippocampus, as the firing rate and the temporal pattern of firing that heavily depends on oscillatory activities^49^. This example motivated us to test whether air puffs allow Tk-GAL4^2^ ∩ Vglut neurons to encode a visual object as oscillatory activity. We first validated that our calcium imaging setup can detect oscillatory activities of at least up to 25 Hz (Supplementary Fig. 6a). Strikingly, when we analyzed the power spectra of the calcium imaging data presented in Fig. 5, we discovered that the power of θ (4–8 Hz) and α (8–16 Hz) oscillations transiently increased specifically when a visual object was presented following, but not before, air puffs (Fig. 6a). We ruled out the possibility that such oscillatory activities occurred simply because air puffs were repeatedly presented, by additionally performing the calcium imaging experiments while presenting air puffs/ a visual object in a reverse order, which yielded a similar result (Supplementary Fig. 6b). Quantification of the changes in power from preceding 0.5 s time bin (Fig. 6b–e) confirmed that θ/ α oscillations transiently increased when a visual object was presented following air puffs (Fig. 6c, d). Air puffs per se also slightly increased θ/ α oscillations, but significantly to lesser degrees (Fig. 6c, d). On the other hand, β oscillations slightly increased in response to air puffs per se, while δ oscillations failed to show stimulus specificity (Fig. 6b, e). We observed similar tendencies irrespective of the stimulation schedule (“forward” schedule as illustrated in Fig. 5c and its “reverse” schedule in Supplementary Fig. 6c–j). As θ/ α but not δ/ β oscillations appear to be correlated with the visual aversion, these data suggest that θ and/or α oscillations may be causally related to visual aversion. Interestingly, increased θ oscillation in response to a visual object was specific to the cells activated by air puffs (Supplementary Fig. 6k). This observation hints at the possibility that the fraction of Tk-GAL4^2^ ∩ Vglut neurons which encodes both air puffs and a visual object may be responsible for puff-gated visual aversion.

We next sought to test whether θ/α oscillations in Tk-GAL4^2^ ∩ Vglut neurons causally contribute to visual aversion. If this is the case, photoactivating Tk-GAL4^2^ ∩ Vglut neurons with θ/α frequencies while presenting a visual object, without preceding air puffs, should promote visual aversion more efficiently than other frequencies (Fig. 6f, left). Note this experiment differs from those of Fig. 3e-h and Fig. 4: there, we photoactivated neurons labeled by Tk-Gal4^2^ (Fig. 3e-h) or Tk-GAL4^2^ ∩ Vglut neurons (Fig. 4) before presenting a visual object to mimic the calcium elevation that occurs in response to air puffs; in contrast, in this experiment we photoactivated Tk-GAL4^2^ ∩ Vglut neurons during the presentation of a visual object, to mimic θ/α oscillations that occur in response to a visual object. To this end, we photoactivated Tk-GAL4^2^ ∩ Vglut neurons with different frequencies, while presenting a small visual object without preceding air puffs (Fig. 6f, right). Remarkably, despite that we kept the total amount of delivered photons constant across different frequencies (see Online Methods), photoactivation with a frequency matching θ (6 Hz) most effectively gated visual aversion (Fig. 6g, h; Supplementary Movie 3). In contrast, photoactivation of Tk-GAL4^2^ ∩ Vglut neurons with other frequencies failed to gate visual aversion at statistically significant levels (Fig. 6g, h; Supplementary Movie 3). Theta is therefore more likely than other frequencies to causally contribute to visual aversion.

Is θ oscillation, then, necessary for puff-gated visual aversion? Previous studies have successfully suppressed an oscillatory activity by artificially inducing oscillation of irrelevant frequencies^50–52^. We thus sought to test if forced induction of frequencies other than θ, during visual stimulus, suppresses puff-gated visual aversion (Fig. 6i, left). To this end, we applied air puffs to the fly, and then presented a visual object while photoinducing different frequencies in Tk-GAL4^2^ ∩ Vglut neurons (Fig. 6i, right). Indeed, photoinduction of all but θ frequency (6 Hz) suppressed the puff-gated visual aversion (Fig. 6j). Our data hence suggest a causal link between visually-evoked θ oscillation and puff-gated visual aversion. Overall, we propose a model of the neuronal mechanism underlying puff-gated visual aversion as follows (Fig. 6k): without air puffs, visual information is fed into Tk-GAL4^2^ ∩ Vglut neurons but fails to induce a response; in contrast, air puffs induce a calcium elevation in Tk-GAL4^2^ ∩ Vglut neurons, allowing them to increase θ oscillation upon subsequent visual information, which at least in part contributes to visual aversion.

### Artificial theta oscillation in Tk-GAL4^2^ ∩ Vglut neurons aversively biases an attractive visual bar

**Fig. 7:**
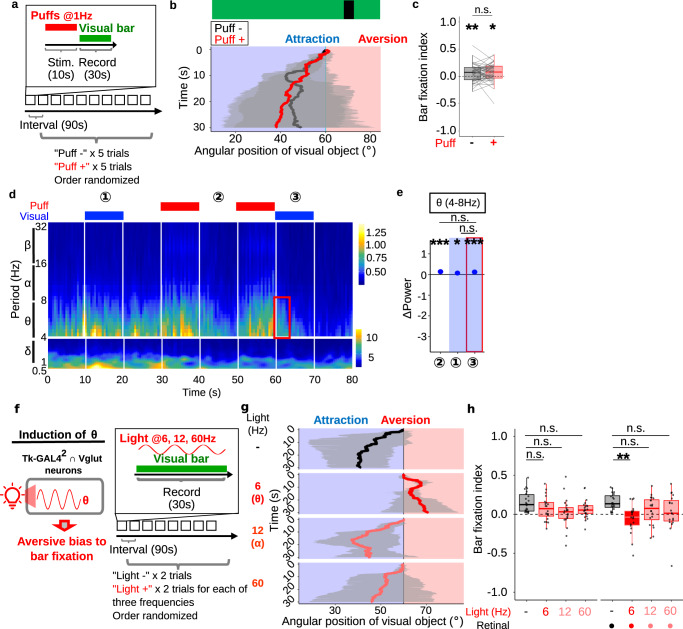
Artificial theta oscillation in Tk-GAL4^2^ ∩ Vglut neurons aversively biases an attractive visual bar. **a** Time course of the experiment. Each fly underwent 10 trials with or without preceding air puffs in a randomized order. **b** Example time courses of a vertical bar’s angular position with or without prior air puffs. For each group, results of all trials of an example fly were pooled. Lines and shaded areas represent means and SEM, respectively. **c** Average bar fixation indices of “puff -“ trials and “puff +“ trials per fly. *N* = 50. In this and following, box plots are generated so that center line indicates median, box limits indicate upper and lower quartiles, and whiskers indicate 1.5x interquartile range. **p < 0.01, *p < 0.05, n.s.: *p* > 0.05, two-tailed *t*-test followed by Bonferroni correction for each puff condition, and two-tailed paired *t*-test for between-conditions comparison. **d** Average wavelet cross-spectrum of the neural recordings. Red rectangle indicates the area of the θ frequency band which was observed when a small object was presented (Fig. 6b). *N* = 98 cells from 7 flies. **e** Power of θ oscillatory activity subtracted by power of the corresponding band during the 0.5 s time bin immediately prior to each time window, for each cell. *N* = 98 cells from 7 flies. ***p < 0.001, *p < 0.05, n.s.: *p* > 0.05. Two-tailed *t*-test followed by Benjamini–Hochberg correction for each condition, two-tailed *t*-test followed by Bonferroni correction for between-conditions comparisons. Dots and error bars represent means and SEM. **f** A schematic of hypothesis. **g** Example time courses of the visual object’s angular position. For each photoactivation frequency, results of all trials of an example fly were pooled. Lines and shaded areas represent means and SEM, respectively. **h** Light-induced changes in the bar fixation indices. *N* = 21, 22, for retinal-unfed and retinal-fed groups, respectively. **p < 0.01, n.s.: *p* > 0.05, two-tailed *t*-test or Wilcoxon signed rank test followed by Benjamini–Hochberg correction.

We next wondered whether gating of θ oscillation and resultant visual aversion are specific to a small object, or instead are induced by other visual objects as well. Flies are widely known to be attracted to a dark vertical bar, which is often referred to as bar fixation^53,54^. We thus examined whether air puffs influence bar fixation, with essentially the same experimental schedule as in Fig. 1h except that a vertical bar was presented instead of a small object (Fig. 7a). As previously reported, flies fixated on a vertical bar before receiving air puffs (Fig. 7b, black line; Supplementary Movie 4). Likewise, when the bar was presented following air puffs, flies showed fixation (Fig. 7b, red line; Supplementary Movie 4). Bar fixation index, which is the inverse of avoidance index, also showed robust attraction with or without air puffs (Fig. 7c). This observation suggests that, in contrast to the response to a small object, air puffs fail to influence bar fixation at a statistically significant level.

Given the causal relationship between θ oscillation and visual aversion, we predicted that a vertical bar would fail to evoke θ oscillation. To test this prediction, we performed a calcium imaging experiment as in Fig. 6, this time presenting a vertical bar instead of a small object. This experiment revealed that a vertical bar indeed fails to evoke θ oscillation even following air puffs (Fig. 7d). Quantification of the changes in power from preceding 0.5 s time bin confirmed that θ oscillation failed to show a larger increase when a vertical bar was presented following air puffs as compared to when a vertical bar alone or air puffs alone were presented (Fig. 7e). This observation is consistent with our data that air puffs fail to gate aversion from a vertical bar at a statistically significant level (Fig. 7a-c).

Having thus established that gating of θ oscillation and the resultant visual aversion take place in response to a small object but not to a vertical bar, we next asked whether forced induction of θ oscillation can aversively bias bar fixation (Fig. 7f, left). To this end, we optogenetically induced θ oscillation while presenting a vertical bar, and recorded the fly’s behavior (Fig. 7f, right). Remarkably, photoactivation with a frequency matching θ aversively biased the bar fixation in the retinal-fed test group (Fig. 7g, h), while frequencies other than θ (6 Hz) in the retinal-fed test group failed to do so at statistically significant levels (Fig. 7g, h). Statistically insignificant reduction in bar fixation observed across different frequencies in the retinal-unfed control group (Fig. 7h) may be attributed to the red light not entirely undetectable to the fly^55^. Together, our data suggest that even bar fixation, which is unaffected by air puffs, can be aversively biased by artificial θ activity in Tk-GAL4^2^ ∩ Vglut neurons.

Altogether, the present study shows that threatened Drosophila gates visual aversion through Tk and a single cluster of Tk-expressing neurons. Our data illustrates how mechanical threats and a visual object can be encoded in the same set of neurons to adapt organismal visual response to a threatening situation.

## Discussion

Here we have revealed that air puffs to fruit flies not only increase locomotion, induce aversion from air puffs, but gate aversion from an otherwise neutral visual object. A single cluster of neurons expressing the neuropeptide Tk is necessary and sufficient for gated visual aversion. A majority of these Tk-GAL4^2^ ∩ Vglut neurons exhibit a calcium elevation upon air puffs. Strikingly, air puffs gate visual response of Tk-GAL4^2^ ∩ Vglut neurons at least in part in the form of θ oscillation, which is causally linked to visual aversion. Collectively, our data suggest a neuronal mechanism in which Tk-GAL4^2^ ∩ Vglut neurons encode both mechanical threats and a visual object to elicit behavioral aversion.

### Air puffs elicit a threat-induced internal state through Tk-GAL4^2^ ∩ Vglut neurons in Drosophila

In the present study, we observed that air puffs increase locomotion (Fig. 1a-f, Supplementary Fig. 1a), induce aversion from air puffs (Supplementary Fig. 1d-f), and gate visual aversion (Fig. 1g-k). We further discovered that Tk and Takr null mutations consistently reduce puff-gated visual aversion (Fig. 2), and that suppression / photoactivation of Tk^+^ neurons reduces / phenocopies puff-gated visual aversion, the latter of which is blocked by Tk null mutations (Fig. 3e-h). In addition, we showed that suppression / activation of Tk-GAL4^2^ ∩ Vglut neurons reduces / phenocopies puff-gated visual aversion (Fig. 4g, h, k, l), although whether the latter is mediated by secretion of Tk molecules remains to be tested. While Tk-GAL4^2^ driver not only labels Tk-GAL4^2^ ∩ Vglut neurons but a group of aggression-inducing neurons^40^, it is unlikely that these neurons contribute to gating visual aversion, because these neurons are located more laterally than Tk-GAL4^2^ ∩ Vglut neurons (Fig. 4f), and are additionally labelled by Tk-GAL4^1^ driver^40^ which appears irrelevant for gated visual aversion (Fig. 3b, c). In addition, unlike aggression-inducing Tk neurons being male-specific^40^, Tk-GAL4^2^ ∩ Vglut neurons exist also in female (Supplementary Fig. 4c), are activated by air puffs (Supplementary Fig. 5b, c), and puff-gated visual avoidance mediated by these neurons in male is also observed in female (Supplementary Fig. 1h), suggesting that the function of Tk-GAL4^2^ ∩ Vglut neurons is largely sex-independent. Compared to male, however, neurite morphology of Tk-GAL4^2^ ∩ Vglut neurons is partially different (Supplementary Fig. 4c) and visual avoidance tended to be slightly weaker (Supplementary Fig. 1h) in female. We therefore do not exclude the possibility that some additional sex-specific regulations may be at work.

While air puffs have historically been used by experimenters as a means to promote locomotion^29,33^, puff responses that we observed in the present study resemble the characteristics of threat responses in mammals^56^, which manifests itself as similar behavioral^1–4^ and cognitive^57,58^ components. Such similarities hint at the possibility that air puffs, serving as mechanical threats, induce a threat-induced internal state in Drosophila. Indeed, a previous literature has suggested that the mechanically increased locomotion may represent an internal state, even with a negative “affective valence”, in Drosophila^36^, on the ground that mechanical threats increase locomotion with scalability and persistence^36^, olfactory sensitization^36^, and release of an odorant that repels other flies^59^. It is notable that the “defensive internal state” has been explicitly reported in Drosophila in the context of repeated overhead shadows^35^. Air puffs to Drosophila may thus serve as a model to gain insight into the neuronal mechanisms underlying threat-induced internal state.

Tk has previously been implicated in nociception^60,61^, anxiety^62^, chronic stress responses^63–66^, metabolic stress^67^, locomotion^68^, and more recently aggression^40,69,70^. Ever since the insect homolog of Tk was first identified in locust^71,72^, evolutionary conservation of Tk functions has been pointed out at least in aggression^40^ and nociception^61^. While the role of Tk molecules in puff-gated visual aversion, specifically whether and how it mediates the function of Tk-GAL4^2^ ∩ Vglut neurons, needs further validation, its role in threat-gated visual aversion may also turn out to be evolutionarily conserved.

How might Tk-GAL4^2^ ∩ Vglut neurons communicate with the visual circuitry to regulate visual aversion? Interestingly, Tk-GAL4^2^ ∩ Vglut neurons (Fig. 4f) appear to send neurites to AOTU, an optic glomeruli directly innervated by two types of visual projection neurons (VPNs), LC10 and MC61^26,73,74^. Of these, LC10 selectively responds to a small visual object^75^. One possibility therefore is that LC10 neurons and Tk-GAL4^2^ ∩ Vglut neurons establish a direct connection in AOTU, although other VPNs that respond to a small object but not to a vertical bar^23,76^ may indirectly communicate with Tk-GAL4^2^ ∩ Vglut neurons. Interestingly, LC10a is indirectly gated by the male sexual arousal center comprised of P1 neurons via yet unidentified circuitry^18,77^. It would be interesting in the future to test whether and how Tk-GAL4^2^ ∩ Vglut neurons communicate with LC10 and how such communication compares to P1-LC10 communication. Such insights may further our general understanding of how visual system is recruited and modulated by different internal states.

### Encoding of air puffs and a visual object in Tk-GAL4^2^ ∩ Vglut neurons

In the present study, we revealed that air puffs evoke a calcium elevation in Tk-GAL4^2^ ∩ Vglut neurons (Fig. 5). This activation is largely mediated by antennae (Fig. 5g-k) at least when the puffs are applied frontally. Given that antennae fully account for wind-induced stopping irrespective of the wind direction^48^, antennae may similarly play a major role in the regulation of visual aversion irrespective of the puff direction, but other mechanosensory systems^78^ may be recruited when puffs are applied from the side or back of the fly. Since substituting air puffs by photoactivation with varying frequencies increased visual aversion with similar degrees (Supplementary Fig. 4f, g), Tk-GAL4^2^ ∩ Vglut neurons likely employ a rate coding scheme to encode air puffs. In contrast, we discovered that Tk-GAL4^2^ ∩ Vglut neurons employ a temporal coding scheme to encode visual information. Specifically, we found that Tk-GAL4^2^ ∩ Vglut neurons, when air puffs are applied beforehand, increase the power of θ oscillation in response to a visual object, and that the increased θ causally contributes to visual aversion (Fig. 6, Fig. 7; Supplementary Fig. 6). We additionally observed that air puffs fail to influence bar fixation at a statistically significant level, that the bar fails to evoke θ oscillation in Tk-GAL4^2^ ∩ Vglut neurons, and that artificial θ oscillation in Tk-GAL4^2^ ∩ Vglut neurons can aversively bias bar fixation (Fig. 7). These observation suggest that the θ oscillation in Tk-GAL4^2^ ∩ Vglut neurons is capable of assigning aversive value to a range of visual information, hinting that these neurons may more widely contribute to the value coding of the visual information.

How might θ activity gate visual aversion? Since neurons are particularly sensitive to high temporal densities of spike input, synchronized outputs from sender neurons may better communicate to their receiver neurons^79^. Alternatively, the flow of information from Tk-GAL4^2^ ∩ Vglut neurons to their downstream target may be improved by increased coherence between the two populations, an idea put forth by “communication through coherence” theory^80,81^. Identification of the downstream target of Tk-GAL4^2^ ∩ Vglut neurons would resolve this issue. Interestingly, θ oscillation in amygdala, hippocampus, and prefrontal cortex (PFC) are thought to play roles in mammalian fear^82–91^ and vision^92–95^. However, such hypotheses have so far been supported by mostly correlational studies. Accordingly, whether and how θ oscillation elicits fear- or vision- related behaviors remain largely unknown. The present study demonstrates a causal link between θ oscillation and visual aversion, and genetically identifies Tk-GAL4^2^ ∩ Vglut neurons wherein θ oscillation subserves such a function. Whether θ oscillation in Drosophila subserves similar functions as that in mammals remains an important issue to be resolved in the future, but our paradigm may prove to be a useful model to gain insights into how θ oscillation generally regulates a threat-induced internal state and vision to shape behavior.

Overall, we have discovered a group of Tk-expressing neurons that gate visual aversion but not increased locomotion. We further revealed how mechanical threats and a visual object can be encoded therein by a combination of rate and temporal coding schemes. Because functions of Tk and θ oscillation appear to be highly conserved across animal kingdom, their roles in visual aversion we describe here may also hold true in other animal species. We propose our findings open up an exciting avenue to the interrogation of how threat promotes visual aversion.

## Methods

### Fly stocks

The following strains were obtained from Bloomington Stock Center (Indiana University); UAS-TNT (#28838), UAS-CsChrimson::mVenus (#55135), 10xUAS-mCD8GFP (#32186), UAS-RedStinger (#8547), 20xUAS-IVS-GCaMP6s (#42749), 20xUAS-IVS-jGCaMP7f (#79031), VGluT-LexA (#60314), LexAop-FLP (#55819), VGlut-GAL80 [MI04979] (#60316), UAS-dicer2 (#24646), UAS-Tk RNAi (#25800).

UAS-FRT-stop-FRT-TNT and UAS-FRT-stop-FRT-CsChrimson::mVenus were gifts from Dr. Barry Dickson (The University of Queensland). *ΔTk*^*1*^, *ΔTk*^*2*^, Tk-GAL4^1^, Tk-GAL4^2^, Tk-GAL4^3^ ^40^ were gifts from Dr. David Anderson (California Institute of Technology) with permission of Dr. Kenta Asahina (Salk Institute). CRISPR null mutation lines^39^ were gifts from Dr. Shu Kondo (National Institute of Genetics, Japan). Cha7.4kb-GAL80^96^ and Gad1-GAL80^96^ were gifts from Dr. T. Sakai (Tokyo Metropolitan University). UAS-FRT-stop-FRT-mCD8::GFP was a gift from Dr. Liqun Luo (Stanford University).

Genotypes and sample sizes used in this study are summarized in Supplementary Data 2.

### Fly preparation for behavioral experiments

Flies were maintained on conventional cornmeal-agar-molasses medium under a 9AM:9PM light/ dark cycle at 25 °C and 60 + /− 5% humidity. Flies were collected 0–2 days post eclosion and housed in a group of 9–10 for 2–7 days before testing (except for calcium imaging and optogenetics experiments, in which flies were housed in a group of 4–6). All behavioral experiments were carried out between 2 pm and 9 pm at 25 + /− 0.8 C and 60 + /− 5% humidity.

Immediately before the experiments, flies were briefly (<1 min) cold anesthetized, and their thorax was glued to the rounded tip of a needle with UV bonding glue (Bondic BD-SKCJ) and 405 nm UV light (OSV5XME1C1E, Akizuki electronics. Inc). For each fly, the experiment lasted approximately 45 min and was split into 10 trials unless otherwise stated.

### Behavioral experiments

Behavioral experiments for each fly consisted of acclimation (17 min), calibration (3 min), and visual response tests (25 min), in this order. For each fly, ball tracking record during calibration phase was used to estimate the mean L-R asymmetry per frame. This estimate was used in the following trials throughout to correct for the L-R asymmetry. During visual response tests, mechanical stimulation (given or not) in one trial was chosen in a pseudo-random order so each condition be repeated for 5 times, resulting in the total of 2 × 5 = 10 trials. Inter-trial interval was set to 90 s. A uniform background (80 cd/m^2^) was given throughout the experiment. As for CRISPR screening (Supplementary Data 1), each condition was repeated 3 times; as for optogenetics experiments, each stimulation frequency was repeated twice.

### LED arena

The LED arena consisted of an air-supported foam ball (Yuzawaya, Japan) in a sphere holder. The ball had a diameter of 8 mm. The airflow was constantly monitored with a digital flowmeter (MF-FP10N-H06-010, Horiba estec. Inc) and was adjusted to 500 + /− 10 mL/min. A pair of 850 nm IR LEDs (FRS5CS, Akizuki electronics. Inc) illuminated the fly from the back. A laser tracking sensor isolated from a computer mouse (MA-LSMA4BK, SANWA) was placed behind the ball in the back of the fly. Tracking data were read out by a computer online at ~192 Hz. This allowed online calculation of the instantaneous rotation of the ball. A video camera (MSP-3080, Panrico) was situated above the fly to record behavior at 47 Hz. The video recordings were analyzed offline by AI-based custom python program (elaborated below). Six LED matrices (Medium 16 × 32 RGB LED matrix panel 420, Adafruit) with > 120fps (confirmed by videotaping the matrices with a high-speed camera) surrounded the fly, covering almost the whole visual field of the fly (azimuth, + /− 135°; elevation, + /− 57°; resolution ~2.8°). The visual arena had a luminance of 80 cd m^−2^. The LED matrices were controlled by a custom-written python program based on rpi-rgb-led-matrix package (https://github.com/hzeller/rpi-rgb-led-matrix).

### Visual stimuli

We showed a single black object (22.5° wide, 22.5° high, 0 cd/m^2^) on a green background (80 cd/m^2^). A visual object was placed at either −60° or +60° at random and the fly was then given control of the angular position of the object. As for induction of θ activity in Fig. 6, the fly was given 30 s control of the angular position of the object, because air puffs that would increase the locomotion were not applied in this experiment and thus response to a single black object unfolded more slowly. In Fig. 7, we showed a single black bar instead (22.5° wide, 57° high, 0 cd/m^2^) and the fly was given 30 s control of the angular position of the object, as the bar fixation unfolded relatively slowly compared to response to a small object.

### Mechanical stimulation

Mechanical stimulation was delivered through a glass pipette (tip diameter of ~2 mm), placed ~2 mm in front of the fly, connected with air hose to a compressor (SSPP-3S, Suisaku, Japan) via a solenoid valve (EXA-C6-02C-4, CKD). The solenoid valve was controlled by an Arduino Uno board configured with Arduino IDE (v.1.8.19), which in turn received connection from a desktop computer running the python program which orchestrated the LED matrices, video recording, ball tracking, and puff application. The mechanical stimulation consisted of 10 puffs (except for Supplementary Fig. 1b, j in which the number of air puffs were varied without changing the frequency nor pressure) by alternating the solenoid valve in between 500 ms-open and 500 ms-closed states (1 Hz). The air pressure was measured by digital manometer (HT-1500N, Hodaka Inc.) and was adjusted to 12 kPa.

### Turn estimation and closed-loop walking

Ball tracking data sampled at ~192 Hz was used to estimate the fly’s walking direction and speed. For each frame, change in the virtual x coordinate from the previous frame was calculated, and this value was multiplied by a gain factor. Gain factor was fixed across flies and experiments, and was set as the one that yielded best bar-fixation performance in a separate experiment. The raw ball-tracking data were first down-sampled (interpolated from ~192 to 10 Hz). Flies were excluded from data analysis when the average probability of walking during trials without puff application was over 25%, indicating unusually high arousal. To account for the L-R asymmetry in the tracking data, due in part to the fly not positioned perfectly perpendicular to the tracking sensor, for each fly we tracked the ball for 3 min prior to the closed-loop experiments, and estimated the average asymmetry per frame. This estimate was used in the following trials throughout to correct for the L-R asymmetry.

### Calculation of the avoidance index

We calculated the avoidance index to quantify the avoidance from a small visual object. This index was defined as: {(linear distance along x axis travelled away from a visual object) – (linear distance along x axis travelled toward a visual object)} / {(linear distance along x axis travelled away from a visual object) + (linear distance along x axis travelled toward a visual object)}. The linear distance along x axis was calculated through the air-supported ball’s rotation, as explained in the above paragraph (“Turn estimation and closed-loop walking”). Walk directed away from the small object tended to manifest robustly 2 s after the start of recording (Fig. 1i), and so the recording of 2–5 s was used to calculate the avoidance index. Attraction index was defined as the linear distance along *x*-axis travelled toward the vertical bar divided by the total distance travelled along x axis. For analysis, we rejected flies which showed avoidance index of larger than 0.4 without air puffs/photoactivation, as such high baseline visual aversion would mask the avoidance-promoting effect of air puffs/photoactivation (the percentage of rejected flies in the wild-type experiment (Fig. 1) was 10%, and the percentage stayed around this value throughout the experiments in the present study).

### AI-based identification of behaviors

We analyzed each video offline using our custom neural network program written in python, utilizing python OpenCV package (v.4.2.0). We first cropped each video, shrinking each image from the dimension of 640 × 480 to 260 × 480 so that the fly is located at around the center of each image. A portion of these images, specifically those from wild type flies, were then manually classified into different behaviors or states (flight/ walking/ grooming/ PER/ stopping/ “stuck” (where the air-supported ball is stuck at the edge of the holder so the fly could not move)/ “freeze in air” (fly is detached from the ball and not showing noticeable movements; we rarely observed this state but when we did, it tended to be immediately after puff application)). Each category typically contained ~200 frames from different flies, except for grooming and stuck categories that each contained ~1000 frames. To take advantage of the temporal information, we then generated “mean” images so that, for an image at time t, an average image of time (t–1), t, and (t + 1) was generated, discarding the first and the last frame of each video. This strategy, compared with when we used the original frames, improved the prediction precision by ~8% as estimated by cross validation. These average images were then augmented by adding random modifications in rotation, scale, location, and flip, so that 10 ~ 50 such images were generated from each original image (rotation angles/ scales/ location shifts independently and uniformly sampled (uid) from [–10,10] degrees/ [−10,10]%/ [−10,10]%, respectively). We adjusted the number of augmented images generated from each original image, so that the total number of augmented images does not vary significantly between behavioral categories. Using these augmented images, we trained the network that contained six 3 × 3 convolutional layers and four dense layers. All but the last layer passed through a ReLu activation function and the final layer passed through a softmax function. Each layer was followed by max pooling, with the pool size of 2 × 2 and strides of 2 × 2. To prevent overfitting, dropout of rate 0.2 was implemented following the last four convolutional layers, and dropout of rate 0.3 was implemented following the first three dense layers. The network was trained using Keras with the input frames being a 119 × 150 × 3 patch. During training, the cross-entropy loss between the predicted score-map and the ground-truth score-map was minimized by stochastic gradient descent. The final model was trained with the batch size of 128 and 150 epochs, which achieved 95% of classification precision as estimated by cross validation. After training, the network predicted entire video frames, which were then thresholded so that confidence of >70% was required for the most likely behavior to be designated to a frame. We discarded trials where the labeling rate fell below 70%. We further discarded trials where “stuck” or “freeze in air” accounted for >10% of all successful labels. Also, trials where “flight” accounted for >10% of all successful labels were excluded from analyses concerning visual aversion and walking velocity.

### Antenna manipulations

To test the role of antennae in the detection of air puffs, a3 segments were either surgically removed using a pair of forceps or glued to a2 segment with a small drop of ultraviolet-activated glue. The glue was cured with uv bonding glue (Bondic BD-SKCJ) and 405 nm uv light (OSV5XME1C1E, Akizuki electronics. Inc). Both manipulations were performed on CO_2_-anesthetized flies 2 days before testing. Flies that underwent CO_2_-anesthesia and touching of antennae with a pair of forceps 2 days before testing served as controls.

### Optogenetics

Flies expressing CsChrimson, a red-shifted channelrhodopsin variant, in neurons labeled by Tk-GAL4^2^ or Tk-GAL4^2^ ∩ Vglut neurons were placed on food containing 500 µM all-trans retinal for 3 days prior to testing. Photoactivation was performed by delivering light using a LED light source (617 nm, Thorlabs) through a glass pipette (tip diameter ~1 mm) wrapped with foil, creating a light spot of ~2 mm diameter on the fly’s head. The output for LED was measured with a power meter (S121C and PM100A, Thorlabs) at a position corresponding to the fly’s head, and was adjusted to 1 μW/mm^2^ for Fig. 3e–h and 2.1 μW/mm^2^ for the rest of the experiments. As for neurons labeled by Tk-GAL4^2^, 40 ms pulse light was applied at 25 Hz; as for Tk-GAL4^2^ ∩ Vglut neurons, light was applied at frequencies specified in the figures, with the pulse duration adjusted for each frequency so that light was applied half the stimulation period on average (i.e. 500 ms for 1 Hz, 166.7 ms for 3 Hz, 83.3 ms for 6 Hz, 50 ms for 10 Hz, 41.7 ms for 12 Hz, 20 ms for 25 Hz, and 8.3 ms for 60 Hz). The averages of results of all frequencies are shown in Fig. 4k, l and Supplementary Fig. 4e, but frequency-wise results are also shown in Supplementary Fig. 4f, g. Light stimuli were triggered by python through an Arduino board connected to the LED driver. As for Fig. 4k, l and Supplementary Fig. 4d–g, red light was applied concomitantly with air puffs to the fly whose third segments (a3) of antennae were surgically removed. We took this approach because a3 houses wind-sensing neurons, and a3-less flies tended to increase locomotion without gating visual aversion.

### Immunohistochemistry

The following antibodies were used: mouse nc82 (1:10 Developmental Studies Hybridoma Bank Cat# nc82), goat anti-mouse Alexa 633 (1:100, Molecular Probe #A21050). Whole brain immunohistochemistry was performed as described previously^97,98^. Briefly, brains were dissected out in 0.3% PBST, and were fixed in 2% paraformaldehyde/ PBS for 90 min at room temperature. Brains were then washed in 0.3% PBST, for >3 h, and were blocked in 10% normal goat serum/ 0.3% PBST for 30 min. Brains were incubated in the primary antibodies in 10% normal goat serum/ 0.3% PBST at 4 °C for 2 days. Brains were then washed in 0.3% PBST for >5 h, and were blocked in 10% normal goat serum/ 0.3% PBST for 30 min. Brains were then incubated in the secondary antibodies in 10% normal goat serum/ 0.3% PBST at 4 °C for 2 days. Afterward, brains were washed in 0.3% PBST for >5 h, and incubated in 50% glycerol/ 0.3% PBST for 2 h at 4 °C. Lastly, brains were mounted on slide glass in Vectashield (Vector Laboratories). Images were taken with Leica TCS SP8 confocal microscope (LASX (v1.1.0.12420)), and processed in Image J (NIH, v1.53t) software.

### Calcium imaging

Two-photon calcium imaging was conducted as described previously^99^ with a few modifications. Two-photon microscopy (Bergamo II, Thorlabs) with a 16x objective lens (16x CFI LWP Plan Flour Objective, Nikon) and with near-infrared excitation (930 nm, Mai Tai 2, SpectraPhysics. Inc., Mountain View, CA), along with ThorImage LS (v3.2.2018.4241) and MaiTai (v0250-2.00.23), was used. Cuticle of the top of the flies’ head and the fat and trachea beneath it were surgically removed. The exposed brain was submerged in saline solution. The extracellular saline had the following composition (in mM): 103 NaCl, 3 KCl, 5 HEPES, 10 trehalose, 10 glucose, 7 sucrose, 26 NaHCO_3_, 1 NaH_2_PO_4_, 1.5 CaCl_2_, 4 MgCl_2_.We identified Tk-GAL4^2^ ∩ Vglut neurons using the baseline fluorescence of GCaMP. Although the Gal4 driver line used for this experiment labeled neurons other than Tk-GAL4^2^ ∩ Vglut neurons, soma of Tk-GAL4^2^ ∩ Vglut neurons form a cluster exclusively in the superior medial protocerebrum, allowing us to unambiguously separate these neurons from the rest. jGCaMP7f was used for all experiments but Fig. 5h, i and Supplementary Fig. 5a, in which GCaMP6s was used instead. Images were acquired from 10 planes at 4 frames per s (256 × 256 pixels, pixel size = 0.399 µm, dwell time = 0.154 µs) for GCaMP6s, and 1 plane at 152 frames per s (160 × 96 pixels, pixel size = 0.388 µm, dwell time = 0.246 µs) for jGCaMP7f. During calcium imaging, a large fraction of the fly’s body was glued to the holding plate, and part of the fly’s eye compounds was covered with the holder, and the fly was hung in the air without an air-supported ball. Visual object was presented by equipping the two-photon microscopy with the LED matrices used in the behavioral experiments, with several modifications: first, the background color was blue instead of green, to prevent the background light from interfering with the GCaMP signal. Second, in order to minimize the bleed-through of the blue light to the range of green, the entirety of the fly, fly holder, and the objective lens were covered with foil, but with an opening through which the fly could see the LED matrices. A blue-pass glass filter was attached to this opening, again to minimize the bleed-through of the blue light to the range of green. The blue-pass glass filter was composed of four filters, each measuring 35 × 50 × 2.5 mm (E-B390, HOYA). A pair of filters were stacked to double the thickness (thus measuring 35 × 50 × 5 mm), and were attached perpendicular to its identical counterpart to form a L-shaped structure. This L-shaped filter was placed so that each inner wall of the “L” faces the fly at 45 degrees from left and right. As for the presentation timing of air puffs and a visual object, we implemented a couple of schedules, one with a reversed order of the other, to control for the effect of timing: 1. rest > visual object > rest > puff > rest > puff > visual object > rest, 2. rest > puff > visual > rest > puff > rest > visual > rest. Data of these schedules were pooled for Fig. 6b-e and Supplementary Fig. 6k. Each fly underwent the sequence of stimuli per fly either twice, three times, or four times, depending on the angle of the brain relative to the objective lens, to scan as many Tk-GAL4^2^ ∩ Vglut neurons as possible distributed along z axis. The time courses of ΔF/F_0_ (Fig. 5e, h) were calculated by, for each bin, taking the percent difference from F immediately prior to the onset of the initial puff application (t = 30 for Fig. 5e; t = 5 for Fig. 5h). ΔF/F_0_ was transformed into z score as, for each cell: (ΔF/F_0 [t]_ – average(ΔF/F_0_)) / sd(ΔF/F_0_), where t indicates time, average indicates the average across time, and sd indicates standard deviation. Neurons that increased calcium signals by more than one z-score upon air puffs were deemed puff responders in Supplementary Fig. 5e and Supplementary Fig. 6k. Frequency-wise power for each cell was calculated from z-scores by Morlet wavelet transform, using morlet function provided by dplR package in R. This frequency-wise power was then transformed into frequency-band-wise power by, for each frequency band and time, taking the mean value across frequencies that belong to the frequency band. Δpower (Fig. 6b–e, Supplementary Fig. 6c–k, Fig. 7e) were calculated by, for each neuron and frequency band, averaging the signals in a given time window and by subtracting from those values the average signal of corresponding frequency band of the time bin (0.5 s) preceding the time window. Because the increase in theta power was specific to the neurons that responded positively to air puffs (Supplementary Fig. 6k), we limited the calculation of Δpower (Fig. 6b–e, Supplementary Fig. 6c–j, Fig. 7e) to these neurons.

### Data analyses

Data analyses were performed using our custom-written R and python programs.

### Diagram generation

All diagrams were constructed using Inkscape (v1.2, https://inkscape.org/), PowerPoint (v2301, Microsoft Corporation, Redmond, WA, USA), and Blender (v.2.93.1, https://www.blender.org/).

### Statistics

We did not predetermine the sample sizes. The distribution of normality of each group was assessed using Shapiro-Wilk normality test. Parametric tests (unequal variances *t*-test known as Welch’s *t*-test or one-way ANOVA) were used only when distributions were deemed normal; otherwise, non-parametric tests were used (Wilcoxon signed rank test for one-sample tests, and Wilcoxon rank sum test for two-sample tests, and Kruskal-Wallis H test). All statistical tests were two-sided. We employed Bonferroni correction to correct for two testing, and Benjamini–Hochberg correction for more than two testing. p < 0.05 after corrections for multiple comparison was deemed statistically significant. Neither randomization nor blinding was performed for the group allocation during experiments or data analysis. All box plots are generated so that center line indicates median, box limits indicate upper and lower quartiles, and whiskers indicate 1.5× interquartile range. All the statistical results are summarized in Supplementary Data 3.

### Reporting summary

Further information on research design is available in the Nature Portfolio Reporting Summary linked to this article.

## Supplementary information

Supplementary Information

Description of Additional Supplementary Files

Supplementary Data 1

Supplementary Data 2

Supplementary Data 3

Supplementary Movie 1

Supplementary Movie 2

Supplementary Movie 3

Supplementary Movie 4

Reporting Summary

## Data Availability

Source data are provided with this paper as a Source Data file. The raw calcium imaging data generated in this study have been deposited in the Mendeley Data under accession code 10.17632/kn7csgjbbv.1 (https://data.mendeley.com/datasets/xjknk7wxms/1) or via a request to the corresponding author. Source data are provided with this paper.

## Source data

Source Data

## Code availability

All source codes^100^ used in this paper have been deposited in GitHub under accession code 10.5281/zenodo.8004654 (https://github.com/mtsuji172/puff_to_visual_aversion) or via a request to the corresponding author.
