# Supplementary material for: Threat gates visual aversion via theta activity in Tachykinergic neurons
Source: Nat Commun. 2023 Jul 13;14:3987. doi: 10.1038/s41467-023-39667-z (PMC10345120; doi:10.1038/s41467-023-39667-z)
Supplement: Supplementary file 1 — Supplementary Information [file 41467_2023_39667_MOESM1_ESM.pdf]

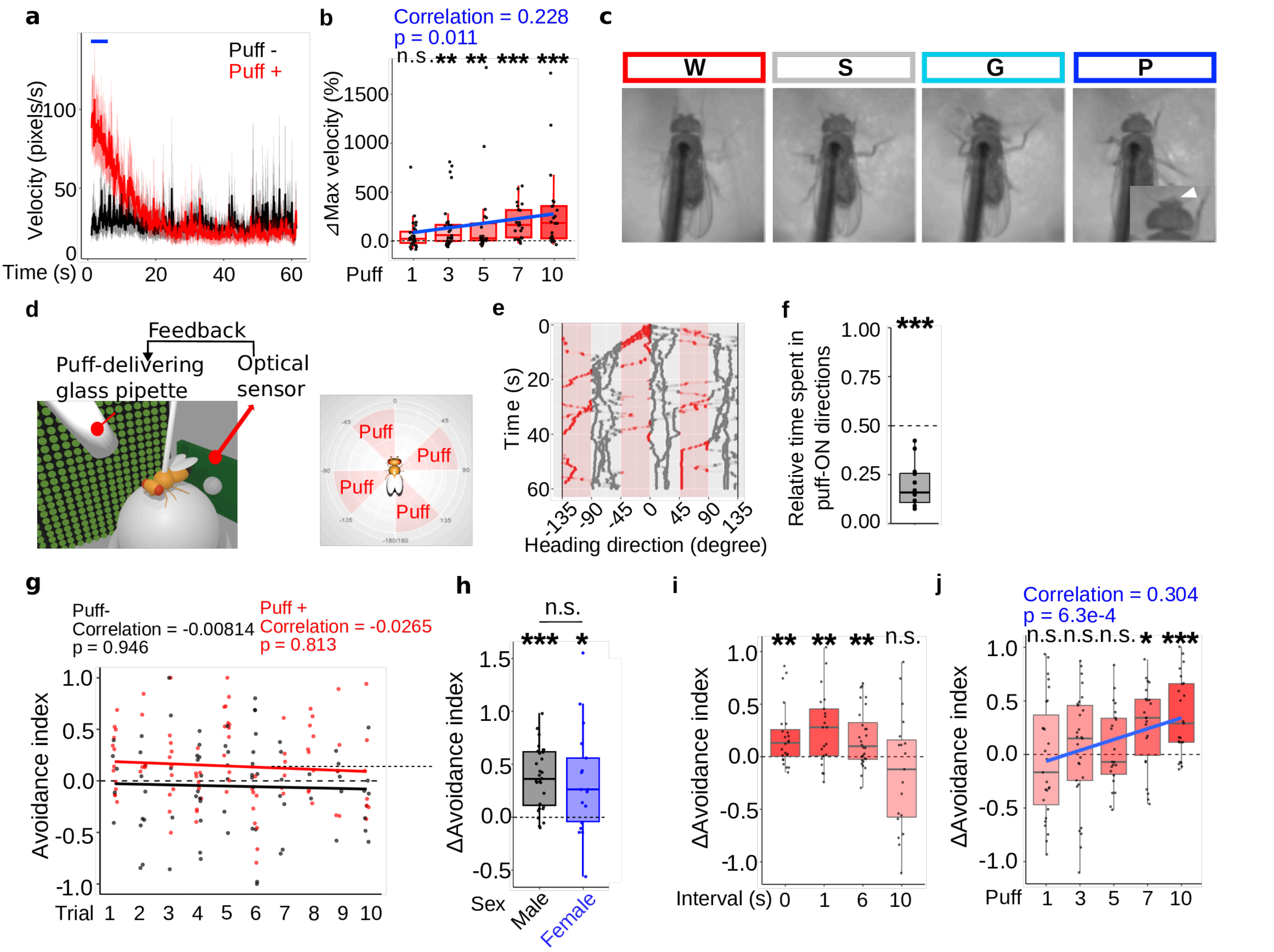

Supplementary Fig. 1 Quantitative characteristics of puff-induced escape responses and visual aversion

(**a**) Time course of velocity. Black/ red lines represent trials without/ with puffs. Lines and shaded regions represent means and SEM. Upper blue line indicates the time window (0-5s) within which maximum velocity was extracted and plotted in **b**. N = 25. (**b**) Change (vs trials without puffs) in maximum velocity against puff number. N = 27, 29, 22, 22, 23 for #puff = 1, 3, 5, 7, 10, respectively. In this and following, box plots are generated so that center line indicates median, box limits indicate upper and lower quartiles, and whiskers indicate 1.5x interquartile range. In this and following, ***p< 0.001, **p< 0.01, *p< 0.05, n.s.: p> 0.05. Two-tailed *t*-test or Wilcoxon signed rank test followed by Benjamini-Hochberg correction. Pearson's product-moment correlation (blue line). (**c**) Example snapshots (average of three consecutive frames) of walking, stopping, grooming, PER of an example fly. Arrowhead in the inlet of PER snapshot shows the extended proboscis in an enlarged view. (**d**) A schematic of the experiment. Virtual puff-ON and OFF angles were tiled for every 45 degrees. While the fly is headed in puff-ON angle, 500ms-puff was applied at 1Hz. (**e**) Traces of all flies. N = 12. (**f**) Ratio of time spent in puff-ON angles. N = 12. Two-tailed *t*-test. (**g**) The avoidance index across 10 trials repeated for each fly. Each dot represents avoidance index of one trial. Lines represent correlations. Black/ red colors represent trials without/ with puffs, respectively. N = 26. Pearson's product-moment correlation (blue line). (**h**) Changes in avoidance index of males and females. N = 30, 17, for male, female. Two-tailed *t*-test followed by Bonferroni correction for each sex, and two-tailed *t*-test for comparison between sexes. (**i**) Changes in avoidance index against post-puff intervals. N = 24, 21, 27, 20 for 0, 1, 6, 10s, respectively. Two-tailed *t*-test or Wilcoxon signed rank test followed by Benjamini-Hochberg correction. (**j**) Changes in avoidance index against puff number. N = 27, 29, 22, 22, 23 for puff(s) of 1, 3, 5, 7, 10, respectively. For each puff number, two-tailed *t*-tests followed by Benjamini-Hochberg correction. Pearson's product-moment correlation (blue line).

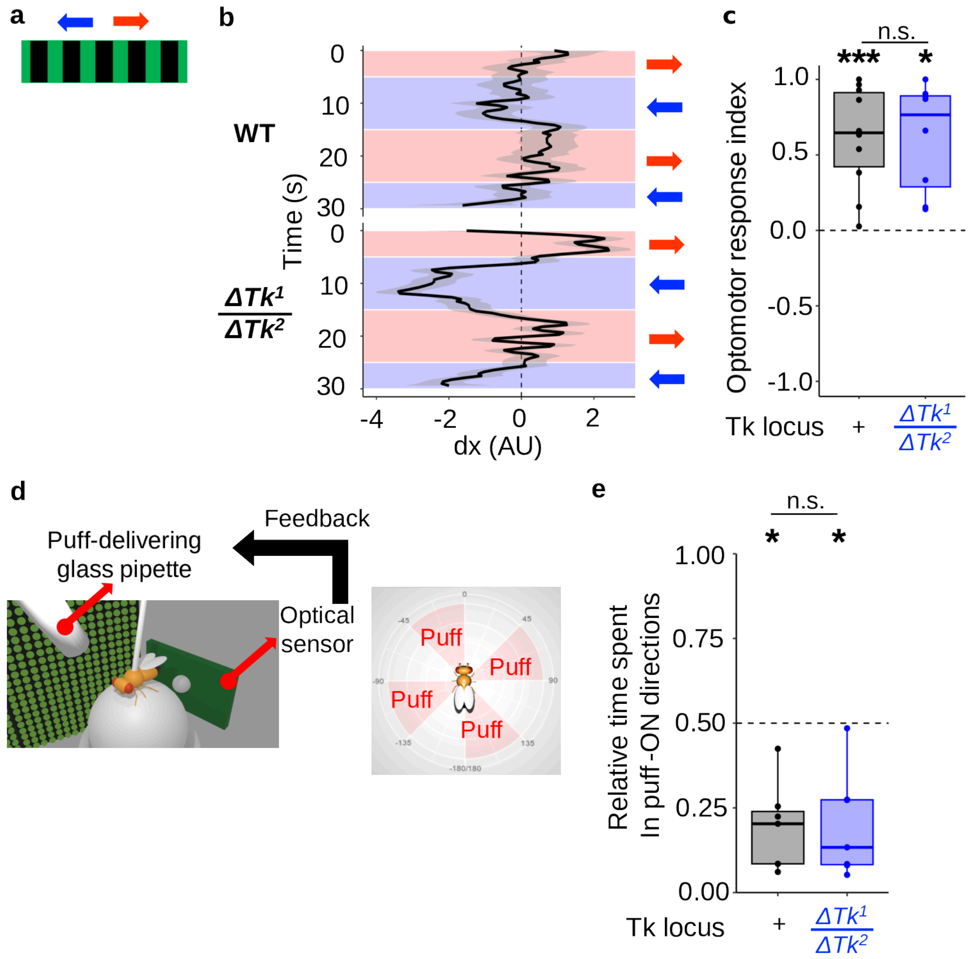

Supplementary Fig. 2 Tk mutant flies retain normal optomotor response and avoidance of air puffs

(**a**) Rotating vertical stripes that changed the direction periodically were presented in an open-loop setting. (**b**) Example time courses of the fly's movement along x axis. For each group, results of all trials of an example fly were pooled. Lines and shaded areas represent means and SEM, respectively. (**c**) Mean position of the visual object throughout the recording period. N = 10, 10, for WT, $\Delta Tk^1/\Delta Tk^2$, respectively. In this and following, box plots are generated so that center line indicates median, box limits indicate upper and lower quartiles, and whiskers indicate 1.5x interquartile range. ***$p < 0.001$, *$p < 0.05$, n.s.: $p > 0.05$, two-tailed *t*-test followed by Bonferroni-correction for each genotype, and two-tailed *t*-test for between genotypes. (**d**) A schematic diagram of the experimental setup. Left: An optical sensor tracked the fly's walking direction and velocity to control the air puffs. Right: An array of virtual puff-ON and puff-OFF angles were tiled for every 45 degrees around the fly. Fly's virtual heading angle was calculated on-line by tracking the ball by an optical sensor. While the fly's heading angle remains in the "puff-ON" angle, air puffs were applied at 1Hz. (**e**) Ratio of the time spent in puff-ON angles. N = 7, 7, for WT, $\Delta Tk^1/\Delta Tk^2$, respectively. *$p < 0.05$, n.s.: $p > 0.05$, two-tailed *t*-test followed by Bonferroni correction for within-genotype comparison, and two-tailed *t*-test for between-genotype comparison.

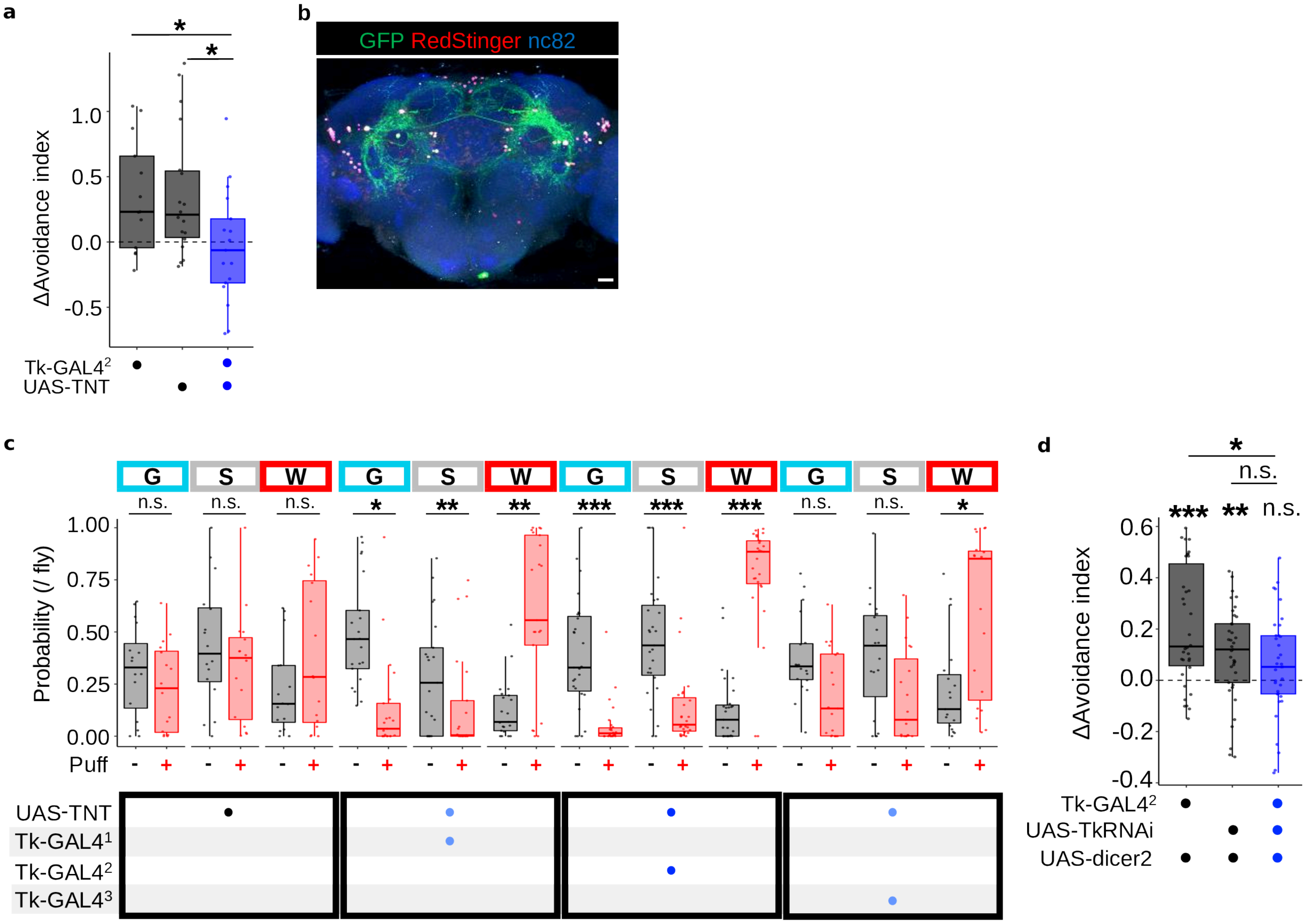

Supplementary Fig. 3 Suppression of Tk$^+$ neurons

(**a**) Puff-induced changes in the avoidance indices. N = 15, 18, 21, for Tk-GAL4^2>+, +>TNT, Tk-GAL4^2>TNT, respectively. In this and following, box plots are generated so that center line indicates median, box limits indicate upper and lower quartiles, and whiskers indicate 1.5x interquartile range. *p < 0.05, two-tailed *t*-test or Wilcoxon rank sum test followed by Bonferroni correction. (**b**) Expression of GFP (green) and RedStinger (nuclear RFP; red) driven by Tk-GAL4^2. Brain was immunostained with anti-neuropil marker nc82 (blue). Scale bar: 25 um. Similar results were obtained across 3 independent samples. (**c**) Probability of each behavior. N = 16, 21, 27, 19, for +>TNT, Tk-GAL4^1>TNT, Tk-GAL4^2>TNT, Tk-GAL4^3>TNT, respectively. ***p < 0.001, **p < 0.01, *p < 0.05, n.s.: p > 0.05, two-tailed paired *t*-test or Wilcoxon signed rank test followed by Benjamini-Hochberg correction. (**d**) Puff-induced changes in the avoidance indices. Precise genotypes are, first: UAS-dicer2 / Y; +; Tk-GAL4^2 / +; second: UAS-dicer2 / Y; +; UAS-Tk RNAi / +; third: UAS-dicer2 / Y; +; Tk-GAL4^2 / UAS-Tk RNAi. N = 34, 37, 35 from left to right, respectively. ***p < 0.001, **p < 0.01, *p < 0.05, n.s.: p > 0.05. Two-tailed *t*-test or Wilcoxon signed rank test followed by Benjamini-Hochberg correction for each genotype, two-tailed *t*-test or Wilcoxon rank sum test followed by Bonferroni correction for comparisons between genotypes.

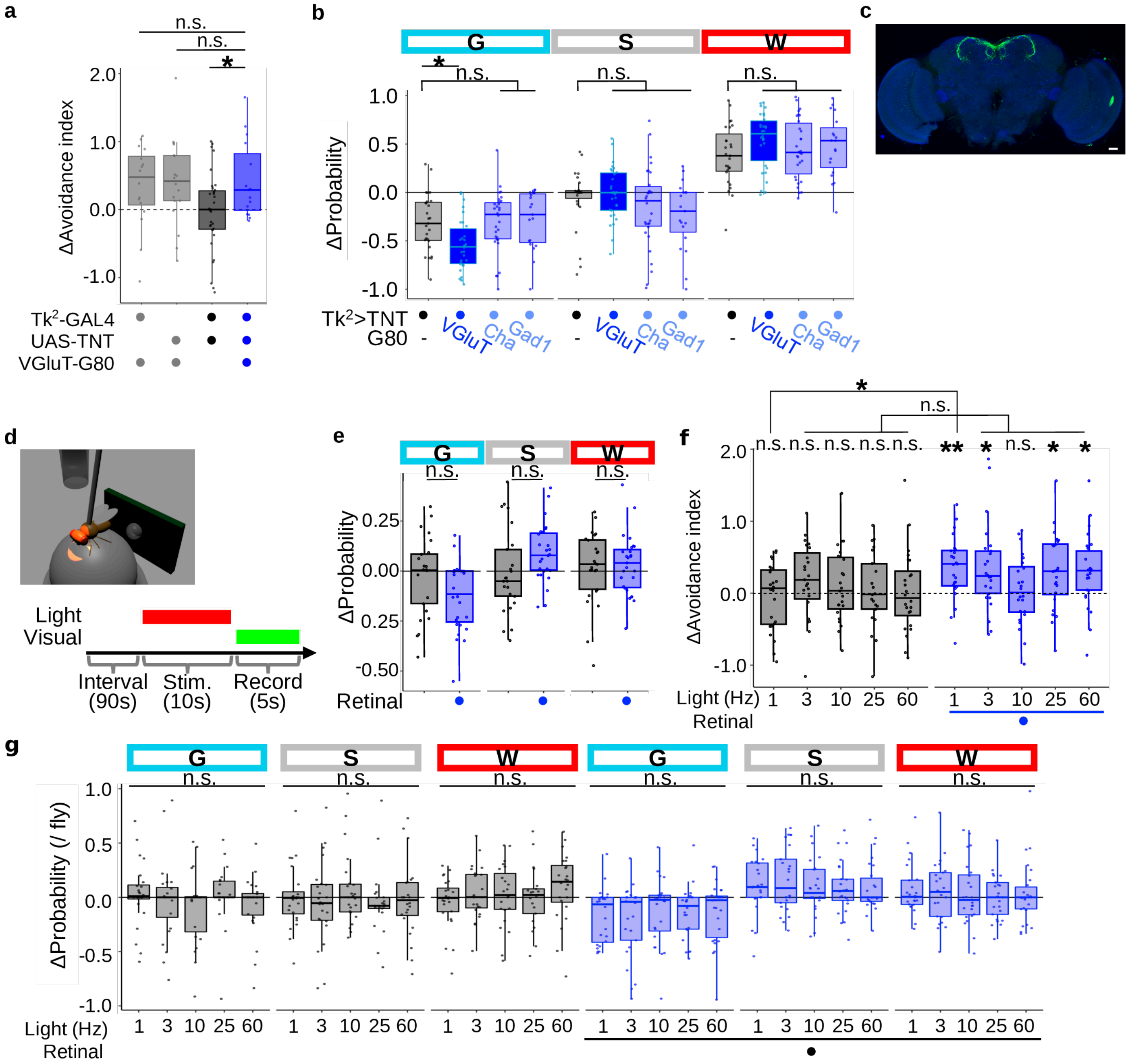

Supplementary Fig. 4 Tk-GAL4[2] ∩ Vglut neurons are responsible for gating visual aversion but not for increasing locomotion, irrespective of their activity frequency

(**a**) Puff-induced changes in the avoidance indices. N = 16, 18, 35, 15, from left to right. In this and following, box plots are generated so that center line indicates median, box limits indicate upper and lower quartiles, and whiskers indicate 1.5x interquartile range.*p < 0.05, n.s.: p > 0.05. two-tailed *t*-test followed by Benjamini-Hochberg correction. (**b**) Puff-induced changes in the probability of grooming, stopping, and walking. N = 27, 30, 30, 19, for GAL80 = -, Cha, VGluT, and Gad1, respectively. *p < 0.05, n.s.: p > 0.05, two-tailed *t*-test followed by Benjamini-Hochberg correction. (**c**) A female brain of VGluT>FLP and Tk-GAL4[2]>stop>mCD8::GFP (green) immunostained with anti-neuropil marker nc82 (blue). Scale bars: 15 um. Similar results were obtained across 2 independent samples. (**d**) A schematic of photoactivation experiments. Red light was shined to the head of the fly to activate CsChrimson expressed in Tk-GAL4[2] ∩ Vglut neurons. (**e**) Puff-induced changes in the probability of each behavior. N = 25, 27, for retinal -, +, respectively. n.s.: p > 0.05, two-tailed *t*-test followed by Benjamini-Hochberg correction. (**f**) Puff-induced changes in the avoidance indices plotted against the photoactivation frequencies. N = 25, 27, for retinal -, +, respectively. **p < 0.01, *p < 0.05, n.s.: p > 0.05, two tailed *t*-test or Wilcoxon signed rank test followed by Benjamini-Hochberg correction for each frequency of retinal - and + groups, two tailed *t*-test or Wilcoxon rank sum test followed by Benjamini-Hochberg correction for comparisons between retinal - and + groups for each frequency. (**g**) Puff-induced changes in the probability of each behavior. N = 25, 27, for retinal -, +, respectively. n.s.: p > 0.05, Kruskal-Wallis test.

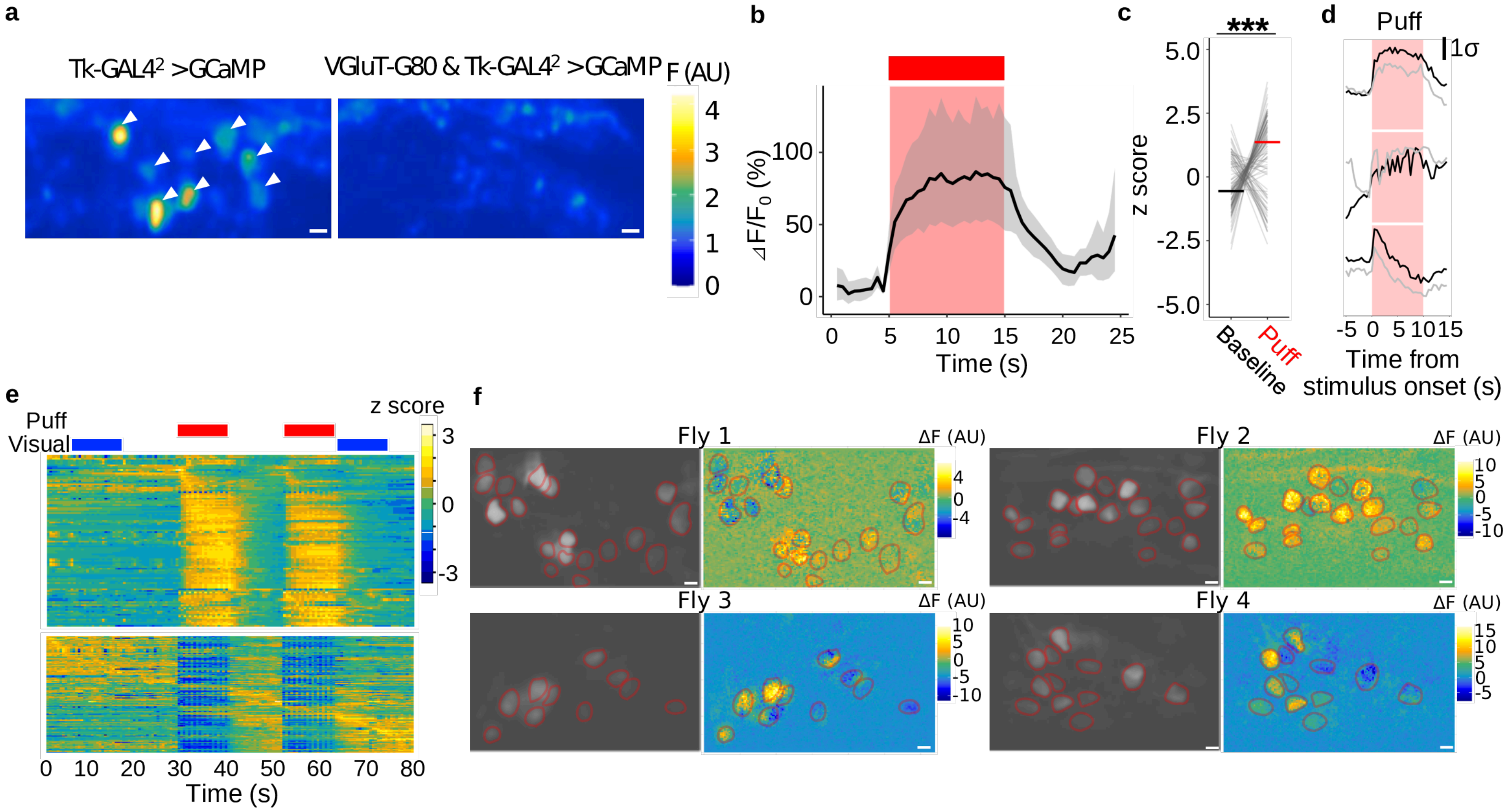

Supplementary Fig. 5 Calcium imaging of Tk-GAL4^2 ∩ Vglut neurons

(**a**) Left: Field of view in an imaged fly expressing GCaMP in neurons labeled by Tk-GAL4^2. The fly is resting without external stimulation, showing GCaMP baseline signals. Fluorescent signal intensity is color-coded. This field of view corresponds to the region occupied by Tk-GAL4^2 ∩ Vglut neurons. Arrowheads indicate the cell bodies. Right: Same as the left image except that this fly additionally carries VGluT-GAL80. Scale bars: 25 um. Similar results were obtained across 13, 10 independent samples for +, VGluT-GAL80, respectively. (**b**) Time course of the $\varDelta F/F_0$ (%) in females. Lines and shaded areas represent means and SEM, respectively. N = 100 cells from 7 animals. (**c**) Calcium response evoked by air puffs in females. The maximal z-scored $\varDelta F/F_0$ values after stimulus onset were averaged. Baseline represents $\Delta F/F_0$ of the frame immediately prior to stimulus onset. Lines represent medians. ***p< 0.001, two-tailed Wilcoxon singed rank test, p< 0.001. N = 100 cells from 7 animals. (**d**) Responses of example cells that were activated on repeated applications of air puffs (30-40s and 60-70s). Black indicates the response during the first application, and gray indicates the second application. (**e**) Rows depict the mean normalized activity of a cell. Red/ blue bars each indicates the time window during which air puffs (30-40s & 50-60s)/ a small object (10-20s & 60-70s) were presented. Neurons which showed increase of larger than 1 z-score upon air puffs are represented in the upper panel, and the rest is represented in the lower panel. (**f**) Field of views in four flies expressing GCaMP in neurons labeled by Tk-GAL4^2. Left: Average image of the whole of 80s recording. Right: Average of the initial puff window (10s), subtracted by the average of the preceding window (10s). Values of each pixel are color-coded. Red circles indicate cell bodies. Scale bars: 25 um. The top, bottom, right, left sides in each figure correspond to the anterior, posterior, left, right sides of the fly's head, respectively. Similar results were obtained across 23 independent samples.

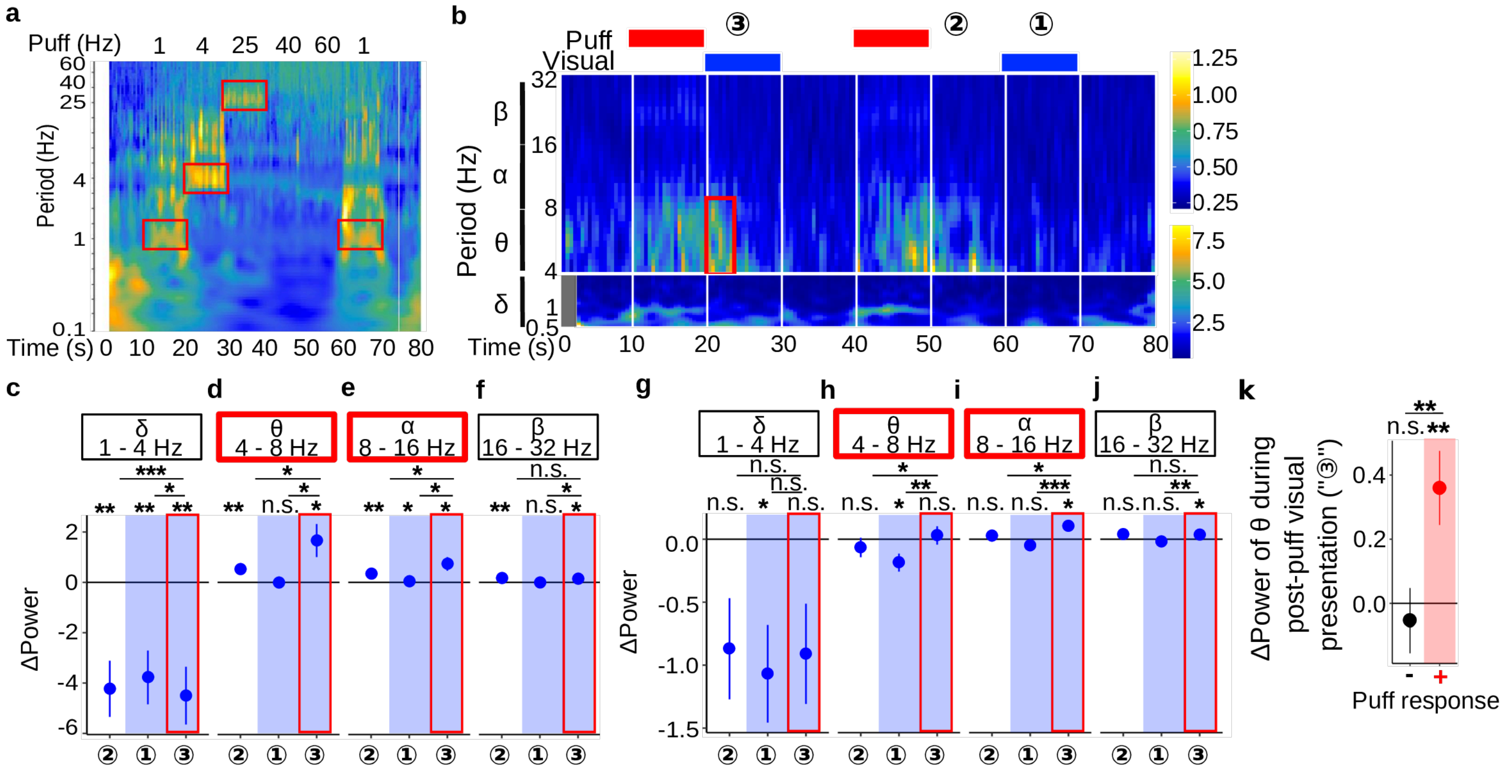

Supplementary Fig. 6 Oscillatory activities of Tk-GAL4^{2} ∩ Vglut neurons

(**a**) The wavelet cross-spectrum of the neural recordings, while air puffs of different frequencies (1, 4, 25, 40, 60Hz, as shown in the upper part of the figures) were applied for consecutive 10 s windows, averaged over all the recorded neurons. Red boxes indicate the frequencies that correspond to the frequency of air puffs applied. (**b**) Average wavelet cross-spectrum of the neural recordings, as in Fig.6a except that the time course of stimuli was reversed in this experiment. Red rectangle indicates the puff-gated visual response in θ/ α frequencies. Note, for frequencies below 4Hz, the initial 2s that contained exceedingly strong signals are masked with gray rectangle. N = 142 cells from 5 animals. (**c** - **j**) Power of δ, θ , α , β oscillatory activities, subtracted by power of the corresponding band during the 0.5s time bin immediately prior to each time window, for each cell. N = 91 cells from 6 flies ("forward": **c** - **f**), N = 60 cells from 4 flies ("reverse": **g** - **j**). ***p < 0.001, **p < 0.01, *p < 0.05, n.s.: p > 0.05. Two-tailed *t*-test followed by Benjamini-Hochberg correction for each window, and two-tailed paired *t*-test followed by Bonferroni correction for comparisons between post-puff visual window and the other windows. Dots and error bars represent means and SEM. (**k**) Power of θ oscillatory activity (averaged across frequencies within θ), subtracted by θ power of the time bin preceding the post-puff visual window ("③" in **a**), for each cell, separating the cells that activated upon air puffs from the rest. N = 329 cells from 11 flies. **p < 0.01, n.s.: p > 0.05, two-tailed *t*-test followed by Bonferroni correction for each response characteristics, and two-tailed *t*-test for between-characteristics comparison. Dots and error bars represent means and SEM.
