# Supplementary material for: Threat gates visual aversion via theta activity in Tachykinergic neurons
Source: Nat Commun. 2023 Jul 13;14:3987. doi: 10.1038/s41467-023-39667-z (PMC10345120; doi:10.1038/s41467-023-39667-z)
Supplement: Supplementary file 2 — Description of Additional Supplementary Files [file 41467_2023_39667_MOESM2_ESM.pdf]

**Description of Additional Supplementary Files**

File Name: Supplementary  Data 1
Description: List of neuropeptide null mutant lines used in this study

File Name: Supplementary  Data 2
Description: List of genotypes and sample sizes used in this study

File Name: Supplementary  Data 3
Description: Statistics of data presented in this study

File Name: Supplementary Movie 1
Description: Air puffs promote visual aversion

File Name: Supplementary Movie 2
Description: Tk-GAL4 $14^2$ ∩ Vglut neurons are activated by air puffs

File Name: Supplementary Movie 3
Description: Photoactivation of Tk-GAL4 $^2$ ∩ Vglut neurons at θ frequency promotes visual aversion

File Name: Supplementary Movie 4
Description: Flies show bar fixation with or without preceding air puffs
