# Supplementary material for: Threat gates visual aversion via theta activity in Tachykinergic neurons
Source: Nat Commun. 2023 Jul 13;14:3987. doi: 10.1038/s41467-023-39667-z (PMC10345120; doi:10.1038/s41467-023-39667-z)
Supplement: Supplementary file 10 — Reporting Summary [file 41467_2023_39667_MOESM10_ESM.pdf]

nature portfolio

Corresponding author(s): Kazuo Emoto

Last updated by author(s): Jun 6, 2023

Reporting Summary

Nature Portfolio wishes to improve the reproducibility of the work that we publish. This form provides structure for consistency and transparency in reporting. For further information on Nature Portfolio policies, see our Editorial Policies and the Editorial Policy Checklist.

Statistics

For all statistical analyses, confirm that the following items are present in the figure legend, table legend, main text, or Methods section.

n/a	Confirmed	
☐	☒	The exact sample size (*n*) for each experimental group/condition, given as a discrete number and unit of measurement
☐	☒	A statement on whether measurements were taken from distinct samples or whether the same sample was measured repeatedly
☐	☒	The statistical test(s) used AND whether they are one- or two-sided<br>*Only common tests should be described solely by name; describe more complex techniques in the Methods section.*
☐	☒	A description of all covariates tested
☐	☒	A description of any assumptions or corrections, such as tests of normality and adjustment for multiple comparisons
☐	☒	A full description of the statistical parameters including central tendency (e.g. means) or other basic estimates (e.g. regression coefficient) AND variation (e.g. standard deviation) or associated estimates of uncertainty (e.g. confidence intervals)
☐	☒	For null hypothesis testing, the test statistic (e.g. F, t, r) with confidence intervals, effect sizes, degrees of freedom and P value noted<br>*Give P values as exact values whenever suitable.*
☒	☐	For Bayesian analysis, information on the choice of priors and Markov chain Monte Carlo settings
☒	☐	For hierarchical and complex designs, identification of the appropriate level for tests and full reporting of outcomes
☐	☒	Estimates of effect sizes (e.g. Cohen's *d*, Pearson's *r*), indicating how they were calculated

Our web collection on statistics for biologists contains articles on many of the points above.

Software and code

Policy information about availability of computer code

Data collection	LASX (v1.1.0.12420), python opencv (v4.2.0), ThorImage LS (v3.2.2018.4241), MaiTai (v0250-2.00.23), Arduino IDE (v.1.8.19), rpi-rgb-led-matrix (https://github.com/hzeller/rpi-rgb-led-matrix)
Data analysis	python (v3.8.10), R (v4.2.1), ImageJ (v1.53t), Inkscape (v1.2), PowerPoint (v2301), Blender (v.2.93.1)<br>Custom codes used for data analyses are deposited to https://github.com/mtsuji172/puff_to_visual_aversion

For manuscripts utilizing custom algorithms or software that are central to the research but not yet described in published literature, software must be made available to editors and reviewers. We strongly encourage code deposition in a community repository (e.g. GitHub). See the Nature Portfolio guidelines for submitting code & software for further information.

Data

Policy information about availability of data

All manuscripts must include a data availability statement. This statement should provide the following information, where applicable:

- Accession codes, unique identifiers, or web links for publicly available datasets
- A description of any restrictions on data availability
- For clinical datasets or third party data, please ensure that the statement adheres to our policy

Source data for all main text and Supplementary Figures can be found in the source data xlsx file provided with this article. The raw calcium imaging dataset is publicly available at https://data.mendeley.com/datasets/xjknk7wxms/1 or via a request to the corresponding authors.

March 2021

Human research participants

Policy information about studies involving human research participants and Sex and Gender in Research.

Reporting on sex and gender	N/A
Population characteristics	N/A
Recruitment	N/A
Ethics oversight	N/A

Note that full information on the approval of the study protocol must also be provided in the manuscript.

Field-specific reporting

Please select the one below that is the best fit for your research. If you are not sure, read the appropriate sections before making your selection.

☒ Life sciences ☐ Behavioural & social sciences ☐ Ecological, evolutionary & environmental sciences

For a reference copy of the document with all sections, see nature.com/documents/nr-reporting-summary-flat.pdf

Life sciences study design

All studies must disclose on these points even when the disclosure is negative.

Sample size	All sample sizes were chosen based on conventional standards used in our field. This value was determined based on the expected magnitude of inter-individual variability, given published results and our own data.
Data exclusions	Criteria for animal exclusion were pre-established. No data were excluded except for flies whose average probability of walking during trials without puff application was over 25%.
Replication	All experiments were repeated at least twice with the same conclusions. Number of biological replicates are stated in each figure.
Randomization	Isogenic Drosophila strains of genotypes to be tested were randomly sampled from the population for experimentation. Covariates were not relevant in this study as experimental and control experiments were performed in parallel, and flies were maintained under identical rearing conditions. Flies were never arbitrarily assigned to treatment groups, and hence there were no experiments in which randomization could have been performed.
Blinding	Blinding was not relevant to this study because experimental flies were generated by and tested by the same investigator.

Reporting for specific materials, systems and methods

We require information from authors about some types of materials, experimental systems and methods used in many studies. Here, indicate whether each material, system or method listed is relevant to your study. If you are not sure if a list item applies to your research, read the appropriate section before selecting a response.

Materials & experimental systems

n/a	Involved in the study	
☐	☒	Antibodies
☒	☐	Eukaryotic cell lines
☒	☐	Palaeontology and archaeology
☐	☒	Animals and other organisms
☒	☐	Clinical data
☒	☐	Dual use research of concern

Methods

n/a	Involved in the study	
☒	☐	ChIP-seq
☒	☐	Flow cytometry
☒	☐	MRI-based neuroimaging

Antibodies

Antibodies used	Mouse anti-nc82 (1:10 Developmental Studies Hybridoma Bank Cat#nc82), goat anti-mouse Alexa 633 (1:100, Molecular Probe #A21050)

Validation

The anti-nc82 antibody (DSHB) is a standard in the field as a background stain that labels presynaptic active active zones. This antibody has been validated by the manufacturer to be reactive in Drosophila and Mosquito immunohistochemistry. The secondary antibody used for neuropil staining (goat anti-mouse Alexa 633 (Molecular Probe)) is likewise a standard in the field.

Animals and other research organisms

Policy information about studies involving animals; ARRIVE guidelines recommended for reporting animal research, and Sex and Gender in Research

Laboratory animals

Drosophila melanogaster Canton-S wildtype and transgenic strains were used across all experiments. Flies were collected 0 – 2 days post eclosion and housed in a group of 9 - 10 for 2 - 7 days before testing (except for calcium imaging and optogenetics experiments, where flies were housed in a group of 4 - 6; labeled in below list as "*"). The complete list of transgenic strains used are below:
ΔTk1 / ΔTk2
ΔTk1 / ΔTk1
ΔTk2 / ΔTk2
Takr86C
Takr99D
UAS-TNT / +
Tk-GAL41 / Y; UAS-TNT / +
UAS-TNT / +; Tk-GAL42 / +
UAS-TNT / Tk-GAL43
UAS-CsChrimson / +; Tk-GAL42 / +
UAS-CsChrimson / +; Tk-GAL42, ΔTk2 / ΔTk1
UAS-TNT/ VGlut-GAL80 [MI04979]; Tk-GAL42 / +
UAS-TNT/ +; Tk-GAL42 / Cha7.4kb-GAL80
UAS-TNT/ +; Tk-GAL42 / Gad1-GAL80
UAS>stop>TNT / +; Tk-GAL42 / +
VGluT-lexA / +; lexAop-FLP / +
UAS>stop>TNT / VGluT-lexA; Tk-GAL42 / lexAop-FLP
UAS>stop>CsChrimson / Y; VGluT-lexA / +; Tk-GAL42 / lexAop-FLP
*Tk-GAL42 / 20xUAS-IVS-jGCaMP7f
*Tk-GAL42 / 20xUAS-IVS-GCaMP6s
*VGluT-GAL80 [MI04979]; Tk-GAL42 / 20xUAS-IVS-GCaMP6s
UAS-dicer2 / Y; +; UAS-Tk RNAi / +
UAS-dicer2 / Y; +; Tk-GAL42 / +
UAS-dicer2 / Y; +; Tk-GAL42 / UAS-Tk RNAi
10xUAS-mCD8::GFP/ +; Tk-GAL42/UAS-RedStinger

Wild animals

The present study did not utilize wild animals.

Reporting on sex

Male flies were primarily tested in the present study, but sex is clearly reported in the main text, figures, and figure legends wherever female flies are tested (Supplementary Fig. 1h, Supplementary Fig. 4c, Supplementary Fig. 5b,c).

Field-collected samples

The present study did not utilize field-collected samples.

Ethics oversight

This study did not require ethical approval.

Note that full information on the approval of the study protocol must also be provided in the manuscript.
